# Positive associations between mean ambient temperature and involuntary admissions to psychiatric facilities

**DOI:** 10.1192/j.eurpsy.2024.1800

**Published:** 2025-01-10

**Authors:** Noah L. Joore, Marte Z. van der Horst, Eric O. Noorthoorn, Jurriaan F.M. Strous, Fleur J. Vruwink, Sinan Guloksuz, Peter C. Siegmund, Jurjen J. Luykx

**Affiliations:** 1 Department of Psychiatry, University Medical Center Utrecht, Utrecht, The Netherlands; 2 Brain Centre Rudolf Magnus, University Medical Center Utrecht, Utrecht, The Netherlands; 3 GGNet Community Mental Health Centre, Warnsveld, The Netherlands; 4 Department of Psychology, Radboud University Nijmegen, Nijmegen, The Netherlands; 5 Department of Psychiatry, University Medical Center Groningen, Groningen, The Netherlands; 6 Lentis Community Mental Health Care, Groningen, The Netherlands; 7 Mediant Geestelijke Gezondheidszorg, Enschede, The Netherlands; 8 Department of Psychiatry and Neuropsychology, School for Mental Health and Neuroscience, Maastricht University Medical Center, Maastricht, The Netherlands; 9 Department of Psychiatry, Yale School of Medicine, New Haven, CT, USA; 10 KNMI Royal Netherlands Meteorological Institute, Department of Weather and Climate Services, De Bilt, The Netherlands; 11 Mood, Anxiety, Psychosis, Stress & Sleep Program, Amsterdam Neuroscience Research Institute, Amsterdam, The Netherlands; 12 Mental Health Program, Amsterdam Public Health Research Institute, Amsterdam, The Netherlands; 13 GGZ inGeest Mental Health Care, Amsterdam, The Netherlands; 14 Department of Psychiatry, Amsterdam University Medical Center, Amsterdam, The Netherlands.

**Keywords:** ambient temperature, climate change, generalized additive models, involuntary admissions, mental health, meteorological variables, psychiatry

## Abstract

**Background:**

Temperature increases in the context of climate change affect numerous mental health outcomes. One such relevant outcome is involuntary admissions as these often relate to severe (life)threatening psychiatric conditions. Due to a shortage of studies into this topic, relationships between mean ambient temperature and involuntary admissions have remained largely elusive.

**Aims:**

To examine associations between involuntary admissions to psychiatric institutions and various meteorological variables.

**Methods:**

Involuntary admissions data from 23 psychiatric institutions in the Netherlands were linked to meteorological data from their respective weather stations. Generalized additive models were used, integrating a restricted maximum likelihood method and thin plate regression splines to preserve generalizability and minimize the risk of overfitting. We thus conducted univariable, seasonally stratified, multivariable, and lagged analyses.

**Results:**

A total of 13,746 involuntary admissions were included over 21,549 days. In univariable and multivariable models, we found significant positive associations with involuntary admissions for ambient temperature and windspeed, with projected increases of up to 0.94% in involuntary admissions per degree Celsius temperature elevation. In the univariable analyses using all data, the strongest associations in terms of significance and explained variance were found for mean ambient temperature (*p* = 2.5 × 10^−6^, Variance Explained [*r* ^2^] = 0.096%) and maximum ambient temperature (*p* = 8.65 × 10^−4^, *r* ^2^ = 0.072%). We did not find evidence that the lagged associations explain the associations for ambient temperature better than the direct associations.

**Conclusion:**

Mean ambient temperature is consistently but weakly associated with involuntary psychiatric admissions. Our findings set the stage for further epidemiological and mechanistic studies into this topic, as well as for modeling studies examining future involuntary psychiatric admissions.

## Introduction

In recent years, the profound and far-reaching impacts of climate change have become increasingly evident across the globe. Rising temperatures, extreme weather events, sea-level rise, and damaged ecosystems are among the most visible manifestations. Climate change also carries effects on human health, ranging from cardiovascular [1] to mental health [2]. For mental health outcomes, the main meteorological variable of interest in research settings has been ambient temperature [3]. Rising ambient temperatures have been associated with a decline in overall mental health [4], elevated suicide rates [4–6], more frequent emergency department visits, more hospitalizations for psychiatric reasons [4, 7], and more violence in psychiatric facilities [8] and communities [9].

To facilitate adequate healthcare responses and the development of effective policies in the future, it is crucial to dissect the potential impact of climate change on a range of mental health outcomes. Of particular relevance in this context are involuntary admissions to psychiatric institutions, owing to their high impact on patients’ well-being and their strong associations with severity of illness, duration of hospitalization, and quality of life [10], as well as their associated medical and societal costs [11].

Only few studies have examined associations between meteorological variables and involuntary admissions. Two such endeavors, one conducted in Greece [12] and one in Italy [13], resulted in significant, positive associations detected between maximum and mean ambient temperature and involuntary admission cases, but the data for both studies included a single psychiatric hospital, the number of weather stations was limited to one geographical location, perceived temperature was not included as a variable, and statistical models did not account for nonlinear associations. Furthermore, both data samples were from Mediterranean regions, limiting generalizability to other geographical areas. In those studies, additional meteorological factors (such as relative humidity, windspeed, and precipitation) were also found to be associated with the number of involuntary admissions, albeit only in cluster analyses with maximum temperature, the primary dependent variable of interest in both Mediterranean studies.

Given the aforementioned knowledge gaps, we reasoned that studying associations between ambient temperature and involuntary admissions in a Northern European country would be timely. Thus, to assess how meteorological factors may be linked to involuntary admissions, we set out to collect incidence data of involuntary admissions across almost all major psychiatric institutions in the Netherlands, covering all provinces (see Supplementary Figure 1). Then, we retrieved detailed meteorological data from the respective geographical locations of these institutions. Finally, we ran non-parametric association analyses examining associations of several meteorological factors with involuntary admissions.

## Methods

### Outcomes

Data on acute involuntary admissions were retrieved from the Argus register [14]. The Argus register contains, among other data, information on the occurrence of involuntary admissions to psychiatric institutions in the Netherlands. Here, data on the occurrence of involuntary admissions in 23 different psychiatric institutions in the Netherlands between 2012 and 2014 were used. As only two institutions were not included, this data covered involuntary mental health care for 94% of the Dutch population. For 14 institutions, data were available from 2012 to 2014; for 8 institutions, data were available from 2013 to 2014; and for 1 institution, data were available for 2014 only. From 2012 to 2014, the Argus data encompassed a far wider range of institutions compared to other times, with large amounts of data available, rendering it the most suitable period for analysis in this study. Acute involuntary admissions (“Inbewaringstelling,” IBS) are hospitalizations mandated by Dutch psychiatrists in the event of a psychiatric disorder being directly causally related to immediate threats, such as physical aggression and suicidality. Psychiatrists can be called upon by police, healthcare personnel, or bystanders, and once they have examined the patient and deem an immediate hospitalization necessary to prevent further damage (with the patient not consenting to admission), they may write a report and advise authorities to have the patient admitted to a psychiatric facility without further delay. Such acute involuntary admissions are executed when the authorities agree and a hospital bed within a psychiatric facility is found for the specific patient. Within a week’s time into the admission, a judge assesses the need for continued involuntary in-hospital care. Importantly, IBSs differ from non-acute involuntary admissions that may be mandated by a judge in the event of psychiatric condition causing harm in the long run, not immediately. These non-acute involuntary admissions are not part of the current project as several weeks may pass between the decision to initiate procedures for involuntary admissions and the actual hospitalization. Besides daily involuntary admissions incidence, no case-specific data were retrieved. All data were anonymized and all procedures were performed in accordance with Dutch medical-ethical standards and permission to carry out this research was granted by the Institutional Review Board of GGNet Mental Health, Warnsveld, The Netherlands. We abided by the 1964 Helsinki declaration. Additionally, all hospitals consented to the use of their data to investigate determinants of coercive measures and involuntary treatment.

### Exposures

All meteorological data used in the current study pertain to daily conditions and were obtained from the open-source database of the Royal Netherlands Meteorological Institute (KNMI). Within this database, daily meteorological measurements are organized per weather station, with each weather station representing an average area of approximately 1,230 km^2^. The Netherlands is covered by a fine-grained set of 34 such weather stations over a surface area of 41,850 km^2^. The collected meteorological data, methods used for collection [15], and their units are specified in Table 1 and the Supplementary Methods. Perceived temperature was calculated using a wind chill formula [16], which combines windspeed and ambient temperature for temperatures below 10°C, and a heat index formula for temperatures above 27°C [17], taking into account both relative humidity and ambient temperature. For temperatures between 10°C and 27°C, relative humidity and windspeed play no significant role for perceived temperature values (see the Supplementary Methods for further details).

**Table 1.** Meteorological variables of interest (for further details, see the KNMI Handbook for the Meteorological Observations (15))

Meteorological variable (unit)	Definition	Measurement
Daily mean temperature (°C)	Average measured temperature per day	Measured with a thermometer 1.5 m above an open plain of grass
Daily maximum temperature (°C)	Highest measured temperature per day	Measured with a thermometer 1.5 m above an open plain of grass
Daily mean relative humidity (percentage of maximum humidity)	Average measured relative humidity per day	Measured with a hygrometer 1.5 m above an open plain of grass
Daily minimum relative humidity (percentage of maximum humidity)^a^	Lowest measured relative humidity per day	Measured with a hygrometer 1.5 m above an open plain of grass
Daily mean windspeed (m/s)	Average measured windspeed per day	Measured with an anemometer 10 m above a flat terrain
Daily sum of precipitation duration (h)	Total sum of precipitation hours per day	Measured with pluviography on the ground in an open area
Daily sum of precipitation (mm)	Total sum of collected precipitation per day	Measured with a pluviometer on the ground in an open area
Daily sum of global radiation (J/cm^2^)	Total sum of measured global radiation per day	Measured by a pyranometer 1.5 meters above an open plain of grass

a This meteorological variable was not included as an exposure, but instead was used in calculations for perceived temperatures.

Mean ambient temperature was the primary temperature variable (primary exposure) as relative to maximum ambient temperature, mean ambient temperature reflects the temperature throughout the day, not merely during peak temperature hours. Regarding the other meteorological variables of interest (secondary exposures), we applied no hierarchy, as we aimed to test in a hypothesis-generating manner which of such variables was associated with involuntary admissions in univariable and multivariable models.

In addition, in light of this argument, mean ambient temperature was included in the multivariable analyses (explained below) instead of maximum ambient temperature.

### Assignment of psychiatric institutions to corresponding weather stations

The assignment of psychiatric institutions to specific weather stations was determined by the geographical proximity of the weather station to the most populous city within the catchment area of the institution (Supplementary Figure 1). For one weather station, “Wilhelminadorp,” there were no meteorological data available until 2018, in which case data from the second nearest weather station (with a distance of 21.4 km to the closest large city) were used. Across all data, the maximum distance between the most populous city and the assigned weather station was approximately 23.5 km.

At the largest psychiatric institution in the Netherlands, “Parnassia,” one of three wards for each involuntary admission was recorded, allowing the division of these admissions between three distinct geographical locations, and subsequently the assignment of each involuntary admission to the closest weather station.

### Data analysis

Statistical analyses were conducted using RStudio, version 4.3.1. The possible differences in descriptive statistics across study years were analyzed using analysis of variance (ANOVA; with the significance threshold set to 0.05 interpreted as significant differences between years).

To assess the nature and robustness of any possible associations between involuntary admissions and meteorological variables, we utilized generalized additive models (GAMs), coupled with an integrated restricted maximum likelihood (REML) approach. GAMs were chosen for their capacity to examine the associations in a non-parametric, unbiased manner, thereby allowing for a nuanced understanding of the associations, without imposing a rigid structural assumption. The use of REML ensured more reliable estimates than the standard generalized cross-validation method, by penalizing overfitting to the specific data and thereby enhancing the generalizability of the data.

To further mitigate the risk of overfitting, thin plate regression splines were incorporated into the GAMs. These thin plate regression splines penalize the second derivative of the fitted function, thereby reducing the flexibility.

The GAMs were set to assume a quasi-Poisson distribution. This assumption was based on both the descriptive involuntary admissions data and the diagnostic plot results of GAMs with a quasi-Poisson assumption (Supplementary Figure 2). Then, we conducted several analyses to examine the associations between meteorological variables and involuntary admissions.

First, to examine for each exposure specifically the possible associations with involuntary admissions, univariable GAMs were run for all meteorological variables (Table 1).

Second, to examine whether associations detected for mean ambient temperature were consistent across all seasons, the data were then categorized into the four traditional meteorological seasons (see the Supplementary Methods) and analyzed for each season separately. This seasonally stratified analysis was also applied to the other meteorological variables.

Third, to explore associations of several meteorological factors with involuntary admissions in a single model, multivariable analyses were then conducted. In these analyses, meteorological variables found to be associated with involuntary admissions in the univariable GAMs at a *p*-value of <0.2 [18], were combined into a multivariable GAM. The same strategy was used for the seasonally stratified multivariable analyses, where for each season the univariable seasonal results determined which meteorological variables were included.

Finally, to examine a possible delay in associations between involuntary admissions after exposure to the meteorological values, a lagged analysis was conducted. Given the time-series structure of the dataset, we conceived lagged temperature variables by temporarily shifting the temperature readings relative to the dates of involuntary admissions, creating a series of seven variables representing lags of up to 7 days. Each of these variables was then individually examined using GAMs to assess the shape of possible lagged associations and establish whether stronger associations were detected relative to the non-lagged GAM results.

For all GAMs, we report three main test statistics: deviance explained, variance explained, and an *F*-statistic. The deviance explained assesses the improvement in model fit after including the independent variables and is usually presented in the context of GAMs. Variance explained rather measures the explanatory value of including the independent variables in the model and is a generally more well-known statistic. Both were included because of this subtle difference in application and interpretation. The *F*-statistic is most commonly used with ANOVAs, but also in the context of GAMs, this statistic is useful. The *F*-value expresses the relevance of the added independent variable to the model: a low *F*-value (e.g., an *F*-value similar to the estimated degrees of freedom of the independent variable) would mean the independent variable has no significant relevance in the model. The significance threshold for all analyses was Bonferroni-corrected for the nine meteorological exposures of interest (*p* < 0.05/9 = 5.55 × 10^−3^).

## Results

### Descriptive statistics

The total dataset included 21,549 days during which a total number of 13,746 cases of involuntary admissions occurred, across 23 facilities over 3 years (Supplementary Table 1). The average yearly number of involuntary admissions per institute was 221 (standard deviation (SD) = 107.0) in 2012, 235 (SD = 114.1) in 2013, and 238 (SD = 122.7) in 2014, which was not different across years (*p* = 0.228). The mean ambient temperature per year was 10.2°C (2012), 9.7°C (2013), and 11.6°C (2014), resulting in a mean of 10.6°C (SD = 6.2) for the entire study duration, with no significant differences between the years (*p* = 0.50). The lowest mean ambient temperature for the entire study duration was −14.8°C, and the highest mean ambient temperature was 28.6°C (Supplementary Table 2).

The data on involuntary admissions resembled a quasi-Poisson distribution (Supplementary Figure 3), with the variance being higher than the mean (Supplementary Table 3).

We list further descriptive statistics of meteorological variables in Supplementary Table 2 and of daily involuntary admissions in Supplementary Table 3.

Distribution graphs for the meteorological variables are displayed in Supplementary Figure 4, whereas distribution graphs for the seasonally stratified data are displayed in Supplementary Figure 5.

### Univariable association analysis between meteorological variables and involuntary admissions

**Figure 1.**
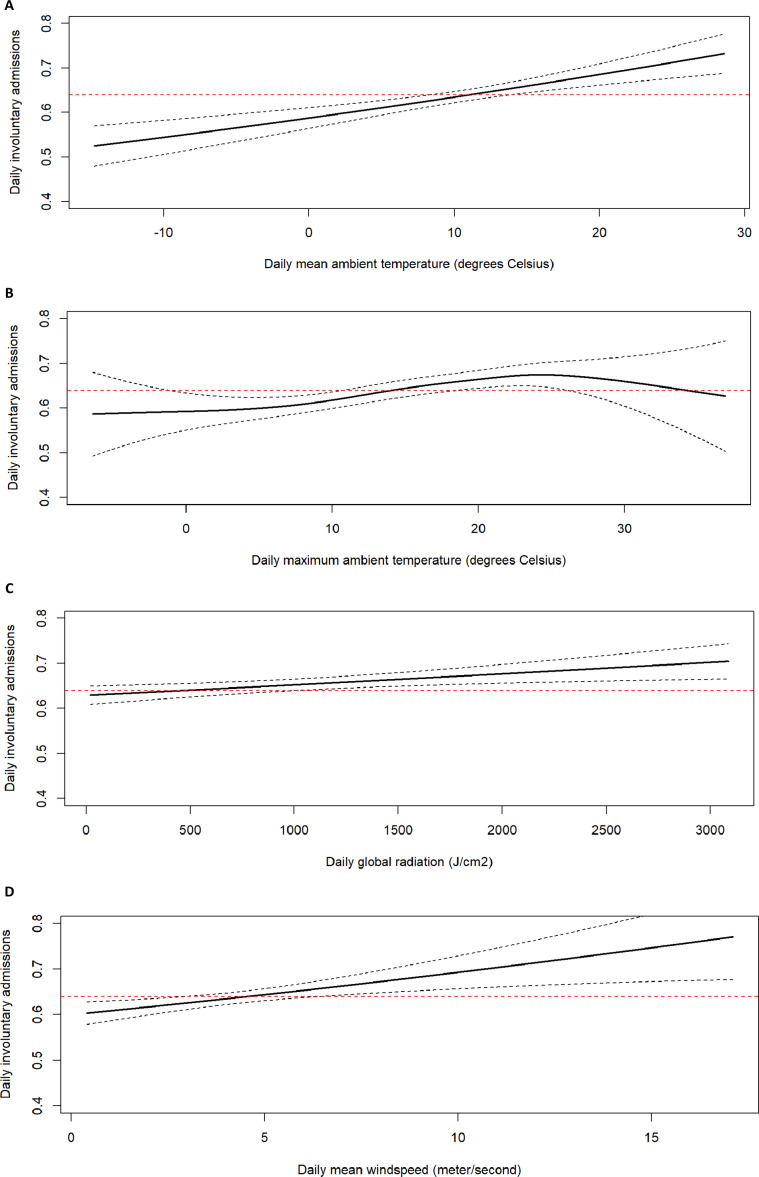
Association plots for the univariable analyses. The *y*-axes represent the expected daily numbers of involuntary admissions according to the generalized additive models we used, averaged per psychiatric institution, and the *x*-axes represent the values of each significantly associated meteorological variable. The dotted black line represents the 95% confidence interval of the association, and the dotted red line represents the mean daily involuntary admissions numbers. (A) Mean ambient temperature (degree Celsius). (B) Maximum ambient temperature (degree Celsius). (C) Global radiation (Joule per square centimeter). (D) Windspeed (meters per second).

In the univariable analyses using all data, we found significant associations of involuntary admissions with the following meteorological variables (in descending order of statistical significance): mean ambient temperature (*p* = 2.5 × 10^−6^, *F*-statistic (*F*) = 22.04, Deviance Explained (DE) = 0.113%, Variance Explained (*r* ^2^) = 0.096%), maximum ambient temperature (*p* = 8.65 × 10^−4^, *F* = 5.01, DE = 0.105%, *r* ^2^ = 0.072%), windspeed (*p* = 1.26 × 10^−3^, *F* = 10.22, DE = 0.0538%, *r* ^2^ = 0.042%), and global radiation (*p* = 4.36 × 10^−3^, *F* = 8.04, DE = 0.0418%, *r* ^2^ = 0.03%) (Figure 1 and Table 2).

**Table 2.** Results with highest significance from the univariable generalized additive model analyses examining associations between meteorological variables and involuntary admissions

Season	Independent variable	*P*-value^a^	*F*-statistic^b^	Deviance explained (%)^c^	Variance explained (%)^d^	EDF^e^
All data	Mean ambient temperature	2.5 × 10^−6^	22.04	0.113%	0.096%	1.015
	Maximum ambient temperature	8.65 × 10^−4^	5.01	0.105%	0.072%	2.949
	Windspeed	1.26 × 10^−3^	10.22	0.0538%	0.042%	1.05
	Global radiation	4.36 × 10^−3^	8.04	0.0418%	0.03%	1.018
Summer	Mean ambient temperature	9.65 × 10^−3^	3.24	0.326%	0.183%	3.327
	Mean perceived temperature	0.012	3.06	0.324%	0.176%	3.426
	Maximum perceived temperature	0.032	2.52	0.293%	0.141%	3.49
Fall	Mean ambient temperature	0.0144	4.22	0.181%	0.121%	1.597
	Windspeed	9.88 × 10^−3^	6.65	0.128%	0.088%	1.004
	Mean perceived temperature	0.022	3.75	0.178%	0.111%	1.774
Winter	Windspeed	0.0308	4.67	0.0944%	0.052%	1.002
	Sum of precipitation	0.192	1.52	0.197%	0.016%	3.221
	Mean relative humidity	0.224	1.46	0.0305%	−0.01%	1.007
Spring	Mean ambient temperature	0.0465	2.26	0.171%	0.081%	2.083
	Maximum ambient temperature	0.053	2.5	0.19%	0.084%	2.457
	Mean perceived temperature	0.066	2.76	0.138%	0.062%	1.793

*Note*: Significant results are presented in bold.

a Indicates the *p*-value.

b Indicates the *F*-statistic.

c Indicates the percentage of deviance explained by the model.

d Indicates the percentage of variance explained by the model.

e Indicates the effective degrees of freedom (EDF) for each meteorological variable.

### Seasonally stratified, univariable association analyses between meteorological conditions and involuntary admissions

In seasonally stratified, univariable analyses, no association met the predefined significance threshold, likely due to the loss of statistical power, as shown in Table 2 and Supplementary Table 5. The same pattern of associations was found for most seasons, with mean ambient temperature being consistently and positively associated with involuntary admissions. The strongest associations for specific seasons were found between mean ambient temperature and involuntary admissions in the summer (*p* = 9.65 × 10^−3^, *F* = 3.24, DE = 0.326%, *r* ^2^ = 0.183%), fall (*p* = 0.014, *F* = 4.22, DE = 0.181%, *r* ^2^ = 0.121%), and spring (*p* = 0.046, *F* = 2.26, DE = 0.171%, *r* ^2^ = 0.081%) data. Supplementary Figure 6 shows the association plots for the most strongly associated meteorological variables for each season.

### Multivariable association analyses between meteorological conditions and involuntary admissions

The multivariable GAM analyses integrated multiple meteorological variables that in univariable GAMs showed a *p* < 0.2, to evaluate their combined association with involuntary admissions. Significant associations with involuntary admissions were found for analyses in all data (DE = 0.21%, *r* ^2^ = 0.15%), summer (DE = 0.21%, *r* ^2^ = 0.23%), fall (DE = 0.45%, *r* ^2^ = 0.30%), and spring (DE = 0.34%, *r* ^2^ = 0.21%) (see Table 3). In these models, the meteorological variables with the strongest associations in terms of significance levels and explained variance were mean ambient temperature in all data (*p* = 1.55 × 10^−3^, *F* = 3.65), summer (*p* = 7.89 × 10^−3^, *F* = 3.35), fall (*p* = 0.019, *F* = 3.39), and spring (*p* = 0.019, *F* = 3.41), and mean windspeed in all data (*p* = 2.67 × 10^−3^, *F* = 10.02), fall (*p* = 6.0 × 10^−3^, *F* = 7.54), and spring (*p* = 0.038, *F* = 3.05) (see Table 3). All results are reported in Supplementary Table 6. When applied to the linear segment of the ambient temperature association (2.5°C–18.5°C), a generalized linear model, examining the association between involuntary admissions and mean ambient temperature, gave a 0.94% rise in involuntary admissions per degree Celsius of temperature elevation (*p* = 1.67 × 10^−5^, *Z*-value = 4.3).

**Table 3.** Results from the multivariable generalized additive model analyses for all data, as well as seasonally stratified data, for each meteorological exposure (variable) included in the models

Season(s)	Meteorological variable	*P*-value^a^	F-statistic^b^	Deviance explained (%)^c^	Variance explained (%)^d^	EDF^e^
All data	Mean ambient temperature	1.55 × 10^−3^	3.65	0.205%	0.149%	3.440
	Windspeed	2.67 × 10^−3^	10.02	0.205%	0.149%	1.011
	Mean relative humidity	0.87	0.01	0.205%	0.149%	1.009
	Global radiation	0.99	0.01	0.205%	0.149%	1.023
Summer	Mean ambient temperature	7.89 × 10^−3^	3.35	0.393%	0.229%	3.346
	Windspeed	0.064	3.43	0.393%	0.229%	1.004
Fall	Mean ambient temperature	0.0185	3.39	0.447%	0.299%	2.322
	Windspeed	6.0 × 10^−3^	7.54	0.447%	0.299%	1.003
	Mean relative humidity	0.57	0.72	0.447%	0.299%	2.456
Spring	Mean ambient temperature	0.019	3.41	0.342%	0.212%	2.092
	Windspeed	0.038	3.05	0.342%	0.212%	2.111
Winter	Sum of precipitation	0.281	1.27	0.239%	0.085%	3.249
	Windspeed	0.166	1.92	0.239%	0.085%	1.003

a Indicates the *P*-value of the smooth function.

b Indicates the *F*-statistic.

c Indicates the percentage of explained deviance by the model.

d Indicates the percentage of explained variance by the model.

e Indicates the effective degrees of freedom (EDF) of the smooth function for the associations.

### Lagged analyses

For the univariable GAM analyses using all data, with lagged “mean ambient temperature” values, all lagged temperatures variables (1–7 lag days) were associated with involuntary admissions, with similar deviances and variances explained as the non-lagged analyses (Supplementary Table 6 and Supplementary Figure 8).

## Discussion

This study, examining associations between meteorological factors and involuntary admissions to psychiatric institutions, included data from 21,549 days across 23 psychiatric facilities over 3 years. Analyses with GAMs were used to flexibly analyze and depict the association strengths and shapes, with GAM-integrated penalization lowering risk of overfitting. Using such univariable models, we first show that ambient temperature is positively and significantly, but weakly associated with involuntary admissions. This association showed the same pattern in all seasonally stratified and multivariable analyses, except for winter temperatures and summer temperatures >18°C, from where a negative deflection of the association was found, albeit with low precision (Supplementary Figure 6).

Of the other meteorological variables, windspeed and global radiation showed positive and significant, but weaker positive associations than mean and maximum ambient temperature. The findings of previous studies, suggesting positive associations of ambient temperature, windspeed, global radiation with involuntary admissions, were based on a single hospital in other geographical regions [12, 13]. The current analyses, combining univariable, multivariable, and stratified models with the use of GAMs, went beyond these studies by taking as a primary independent variable mean ambient temperature, while additionally including a wide range of meteorological variables, during several years across almost all regions of an entire country. Furthermore, we now extend these findings to a different geographical region (Northern Europe) and to a comprehensive set of weather stations and psychiatric institutions, that were geographically closely located. Finally, using both deviance and variance explained as test statistics, we here aimed to give readers a more nuanced understanding of the strength of associations between meteorological variables and involuntary admissions.

Given the novelty of this field of research, determining definite mechanisms underlying the associations we detected is currently challenging. Nonetheless, it is well described that other factors are associated with involuntary hospitalizations to psychiatric institutions, for example, a diagnosis of schizophrenia or substance use disorders, male gender [19], and sociodemographic factors, such as unemployment, lower income, and urban living [20]. Although heat waves increase risk of drought and consequently migration in the long term, effects of heat on migration status are slow and not visible on the same day of admission. Similar reasoning goes for a diagnosis of schizophrenia. It is possible, however, that heat increases chances of substance use disorders becoming apparent, for example, simply because of more time spent outdoors by patients suffering from such disorders, which in turn may result in involuntary admissions. Additional, similarly indirect pathways explaining our findings include reduced sleep quality at higher ambient temperatures [21] and proneness to aggression and violence at higher ambient temperatures [8, 9].

To estimate the possible implications of increasing temperatures on involuntary admissions, a linear model was fitted to the linearly associated temperature data. This model revealed an increase of 0.94% in involuntary admissions prevalence for each degree Celsius increase in temperature. The climate scenarios of KNMI predict a rise in annual mean temperature of 0.9°C–1.6°C in 2050 compared to 1991–2020 [22]. Based on our data, this would mean an extra 39–69 yearly cases of involuntary admissions in the included psychiatric facilities. Our findings may thus help design policies aimed at preparing for and potentially reducing the number of involuntary admissions to psychiatric institutions in the future.

The strengths of this study lie in the unprecedented large amount of data gathered over several years from many psychiatric institutions across the Netherlands (covering almost the entire country), our dense and comprehensive meteorological dataset, and the non-parametric data analysis approach, allowing for detection of linear as well as nonlinear associations, at low risk of overfitting. Limitations include the limited timeframe of the data and the low prevalence of “extreme values” for meteorological variables in the moderate climate of the Netherlands. In addition, the absence of individual-level data, such as age, sex, and psychiatric diagnosis, precluded us from running stratified analyses for specific subgroups of patients. Finally, the explained variance and deviance detected in our models were highly significant and consistent, yet generally low.

In conclusion, we found significant and positive, but weak associations between meteorological conditions (particularly mean ambient temperature) and involuntary admissions in the Netherlands. Our findings set the stage for further epidemiological, climate science and mechanistic studies into this topic, as well as into modeling studies to predict future patterns in involuntary psychiatric hospitalizations.

## Supporting information

Joore et al. supplementary material Joore et al. supplementary material

## Data Availability

The data that support the findings of this study are available from the corresponding author (J.J.L.) upon reasonable request.

## Supplementary material

The supplementary material for this article can be found at http://doi.org/10.1192/j.eurpsy.2024.1800.

## Analytic code availability

A public GitHub repository has been created with the same title as this paper (Association-between-mean-ambient-temperature-and-involuntary-admissions-to-psychiatric-facilities), containing all R-studio functions used in the analyses for this paper.
