# Supplementary material for: Positive associations between mean ambient temperature and involuntary admissions to psychiatric facilities
Source: Eur Psychiatry. 2025 Jan 10;68(1):e2. doi: 10.1192/j.eurpsy.2024.1800 (PMC11795429; doi:10.1192/j.eurpsy.2024.1800)
Supplement: Joore et al. supplementary material [file S0924933824018005sup001.docx]

**Supplement to:**

**Positive Association between Mean Ambient Temperature and Involuntary Admissions to Psychiatric Facilities**

Noah L. Joore*^1^, Marte Z. van der Horst*^1,2,3^, Eric O. Noorthoorn^3,4^, Jurriaan F.M. Strous^5,6^, Fleur J. Vruwink^7^, Sinan Guloksuz^8,9^, Peter C. Siegmund^10^, Jurjen J. Luykx^8,11,12,13^

1 Department of Psychiatry, University Medical Center Utrecht, Utrecht, The Netherlands
2 Brain Centre Rudolf Magnus, University Medical Center Utrecht, Utrecht, The Netherlands
3 GGNet Community Mental Health Centre, Warnsveld, The Netherlands
4 Department of psychology, Radboud university Nijmegen, the Netherlands
5 Department of Psychiatry, University Medical Center Groningen, Groningen, The Netherlands
6 Lentis Community Mental Health Care, Groningen, The Netherlands
7 Mediant Geestelijke Gezondheidszorg, Enschede, Netherlands
8 Department of Psychiatry and Neuropsychology, School for Mental Health and Neuroscience, Maastricht University Medical Center, Maastricht, the Netherlands
9 Department of Psychiatry, Yale School of Medicine, United States of America
10 KNMI Royal Netherlands Meteorological Institute, Weather and Climate Services, De Bilt, The Netherlands
11 Amsterdam Neuroscience (Mood, Anxiety, Psychosis, Stress & Sleep program) and Amsterdam Public Health (Mental Health program) research institutes, Amsterdam, the Netherlands
12 GGZ inGeest Mental Health Care, Amsterdam, The Netherland
13 Department of Psychiatry, Amsterdam University Medical Center, Amsterdam, the Netherland

*Equal contributions

Corresponding author: Dr. Jurjen J. Luykx, j.j.luykx@amsterdamumc.nl.

Table of content:

- Supplementary Methods: p.3
- Supplementary Table 1; Distribution of involuntary admissions per institution per year: p.4
- Supplementary Table 2; Descriptive statistics for meteorological variables: p.5
- Supplementary Table 3; Descriptive statistics for involuntary admission cases: p.5
- Supplementary Table 4; Results of comprehensive, univariable GAM analyses: p.6
- Supplementary Table 5; Results of seasonally stratified, univariable GAM analyses: p.7
- Supplementary Table 6; Results of multivariable GAM analyses: p.8
- Supplementary Table 7; Results of univariable, comprehensive GAM analyses with lagged temperature values: p.9
- Supplementary Figure 1; Map of the assignment of weather stations to psychiatric institutions: p.10
- Supplementary Figure 2; Diagnostic plots of the comprehensive ‘mean daily ambient temperature’ GAM analysis: p.11
- Supplementary Figure 3; Distribution of daily involuntary admission incidence: p.12
- Supplementary Figure 4; Distribution graphs for comprehensive meteorological variables: p.13
- Supplementary Figure 5; Distribution graphs of seasonally stratified data: p.14
- Supplementary Figure 6; Association plots for significantly associated meteorological variables in univariable, seasonally stratified GAMs: p.15
- Supplementary Figure 7; Association plots of significantly associated meteorological variables in multivariable GAMs: p.16
- Supplementary Figure 8; Association plots of lagged GAM analyses between mean ambient temperature and involuntary admissions: p.17
- Supplementary Figure 9; Lambda-estimation plot for temperature-binned involuntary admission cases: p.18
- Bibliography: p. 19

Supplementary Methods

**Perceived temperature variables**

Perceived temperature variables were created by using two formulas:

1. a wind chill formula, specifically the JAG/TI(Geert Groen, 2009) method combining windspeed values and ambient temperature for values below 10 degrees Celsius, and
2. a heat index formula, provided by the National Oceanic and Atmospheric Administration (NOAA) (Brooke Anderson et al., 2013), combining relative humidity and ambient temperature for values above 27 degrees Celsius.

For temperatures between 10 and 27 degrees Celsius, perceived temperature does not significantly differ from ambient temperature.
Thus, two perceived variables were created:

- Mean daily perceived temperature, combining ‘mean daily ambient temperature’, ‘mean daily relative humidity’ and ‘mean daily windspeed’.
- Maximum daily perceived temperature, combining ‘maximum daily ambient temperature’, ‘minimum daily relative humidity’ and ‘mean daily windspeed’. In this case, ‘minimum daily relative humidity’ was used because the time of measurement resembled that of ‘maximum daily ambient temperature’ the most, compared to other relative humidity variables.

**Meteorological data collection**

All meteorological data was collected by the Royal Netherlands Meteorological Institute (KNMI) in accordance with the KNMI Handbook for the Meteorological Observation (Koninklijk Nederlands Meteorologisch Instituut, 2000).

**Stratification of meteorological seasons**

For seasonal stratification, the definition of meteorological seasons from the Royal Netherlands Meteorological Institute (KNMI) was used. This resulted in the following stratification:

- Summer: June 1^st^ – august 31^st^
- Fall: September 1^st^ – November 30^th^
- Winter: December 1^st^ – February 28^th^ / 29^th^
- Spring: March 1^st^ – May 31^st^

Supplementary table 1 – Numbers of involuntary admissions per institution per year

| **Psychiatric institution** | **Number of involuntary admission cases in 2012** | **Number of involuntary admission cases in 2013** | **Number of involuntary admission cases in 2014** |
| --- | --- | --- | --- |
| Altrecht | - | 455 | 440 |
| Arkin | 554 | 272 | 543 |
| Breburg | 208 | 209 | 249 |
| Delfland | 211 | 225 | 247 |
| Dimence | 228 | 270 | 269 |
| Emergis | 160 | 203 | 208 |
| GGnet | 246 | 234 | 165 |
| GGZ Centraal | - | - | 185 |
| GGZ Drenthe | - | 56 | 68 |
| GGZ Eindhoven | 169 | 210 | 170 |
| GGZ Friesland | - | 198 | 208 |
| Lentis | 190 | 193 | 203 |
| Mediant | 203 | 214 | 200 |
| GGZ Noord-Holland Noord | 153 | 131 | 133 |
| Parnassia Haarlem | - | 174 | 156 |
| Parnassia Den Haag | - | 496 | 418 |
| Parnassia Rotterdam | - | 287 | 183 |
| Pro Persona | 130 | 125 | 197 |
| Rivierduinen | - | 467 | 448 |
| Reinier van Arkel | 116 | 136 | 134 |
| Vincent van Gogh | 229 | 251 | 276 |
| GGZ West Noord Brabant | - | 91 | 58 |
| Yulius | 294 | 282 | 318 |

Supplementary Table 2 – Descriptive statistics for meteorological variables

| **Meteorological variable** | **N** | **Minimum** | **Maximum** | **Mean** | **Std. Deviation** |
| --- | --- | --- | --- | --- | --- |
| Daily mean ambient temperature (in degrees Celsius) | 21549 | - 14,8 | 28,6 | 10,59 | 6,17 |
| Daily maximum ambient temperature (in degrees Celsius) | 21549 | - 6,4 | 36,9 | 144,26 | 71,38 |
| Daily mean relative humidity (in percentages) | 21549 | 30 | 100 | 81,38 | 9,37 |
| Daily minimum relative humidity (in percentages) | 21549 | 18 | 100 | 64,86 | 14,59 |
| Daily mean windspeed (in meter/second) | 21549 | 0,4 | 17,1 | 4,34 | 2,22 |
| Daily global radiation (in J/cm^2^) | 20819 | 20 | 3086 | 1027,29 | 770,59 |
| Daily duration of precipitation (in 0.1 hours) | 20819 | 0 | 240 | 17,3 | 28,96 |
|  |  |  |  |  |  |
| Daily sum of precipitation (in 0.1mm) | 20819 | 0 | 1316 | 22,26 | 47,32 |


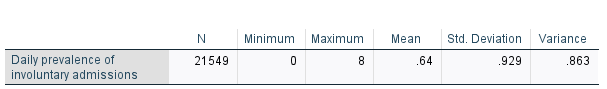
Supplementary Table 3 – Descriptive statistics for involuntary admission cases (“N” refers to the total number of observations across the entire dataset for all years)

|  | **GAM P-value^*^** | **GAM F-statistic^**^** | **Deviance explained (%)^***^** | **Variance explained (%)^****^** | **GAM edf^*****^** |
| --- | --- | --- | --- | --- | --- |
| Daily mean ambient temperature | 2.5*10^-6^ | 22.04 | 0.113% | 0.096% | 1.015 |
| Daily maximum ambient temperature | 8.65*10^-4^ | 5.01 | 0.105% | 0.072% | 2.949 |
| Daily mean relative humidity | 0.194 | 1.68 | 0.0242% | 0.0047% | 1.917 |
| Daily mean windspeed | 0.00126 | 10.22 | 0.0538% | 0.042% | 1.05 |
| Daily global radiation | 0.00436 | 8.04 | 0.0418% | 0.03% | 1.018 |
| Daily duration of precipitation | 0.439 | 0.59 | 0.00322% | -0.0067% | 1.013 |
| Daily sum of precipitation | 0.574 | 0.31 | 0.00198% | -0.008% | 1.024 |
| Daily mean perceived temperature | 1.16*10^-4^ | 17.55 | 0.104% | 0.088% | 1.081 |
| Daily maximum perceived temperature | 0.0013 | 4.41 | 0.108% | 0.07% | 3.361 |

Supplementary Table 4 - Results of univariable GAM analyses in all data

(*) the P-value of the smooth function, (**) the F-statistic of the smooth function, (***) the percentage of explained deviance by the model, (****) the percentage of explained variance by the model and (*****) the effective degrees of freedom of the smooth function for the resulted associations.

Supplementary Table 5 – Results of seasonally stratified, univariate GAM analyses

| **Season** | **Meteorological variable** | **GAM P-value^*^** | **GAM F-statistic^**^** | **Deviance explained (%)^***^** | **Variance explained (%)^****^** | **GAM edf^*****^** |
| --- | --- | --- | --- | --- | --- | --- |
| Summer | Mean ambient temperature | 9,65 * 10^-3^ | 3.24 | 0.326% | 0.183% | 3.327 |
|  | Maximum ambient temperature | 0.0336 | 2.48 | 0.287% | 0.138% | 3.481 |
|  | Mean relative humidity | 0.755 | 0.10 | 0.00212% | -0.035% | 1.004 |
|  | Mean windspeed | 0.0848 | 2.98 | 0.0572% | 0.017% | 1.004 |
|  | Global radiation | 0.359 | 0.82 | 0.0179% | -0.021% | 1.016 |
|  | Duration of precipitation | 0.402 | 0.70 | 0.0143% | -0.024% | 1.001 |
|  | Sum of precipitation | 0.238 | 1.66 | 0.0805% | 0.024% | 1.932 |
|  | Mean perceived temperature | 0.0122 | 3.06 | 0.324% | 0.176% | 3.426 |
|  | Maximum perceived temperature | 0.0323 | 2.52 | 0.293% | 0.141% | 3.49 |
| Fall | Mean ambient temperature | 0.0144 | 4.22 | 0.181% | 0.121% | 1.597 |
|  | Maximum ambient temperature | 0.0286 | 4.78 | 0.0952% | 0.055% | 1.009 |
|  | Mean relative humidity | 0.146 | 1.82 | 0.14% | 0.045% | 2.502 |
|  | Mean windspeed | 9,88 * 10^-3^ | 6.65 | 0.128% | 0.088% | 1.004 |
|  | Global radiation | 0.509 | 0.76 | 0.0392% | -0.022% | 1.579 |
|  | Duration of precipitation | 0.816 | 0.06 | 0.00112% | -0.038% | 1.001 |
|  | Sum of precipitation | 0.562 | 0.34 | 0.00712% | -0.032% | 1.005 |
|  | Mean perceived temperature | 0.0222 | 3.75 | 0.178% | 0.111% | 1.774 |
|  | Maximum perceived temperature | 0.0479 | 3.52 | 0.087% | 0.045% | 1.079 |
| Winter | Mean ambient temperature | 0.416 | 0.66 | 0.0138% | -0.025% | 1.004 |
|  | Maximum ambient temperature | 0.642 | 0.35 | 0.0334% | -0.027% | 1.545 |
|  | Mean relative humidity | 0.224 | 1.46 | 0.0305% | -0.01% | 1.007 |
|  | Mean windspeed | 0.0308 | 4.67 | 0.0944% | 0.052% | 1.002 |
|  | Global radiation | 0.702 | 0.38 | 0.0266% | -0.034% | 1.515 |
|  | Duration of precipitation | 0.591 | 0.57 | 0.0399% | -0.031% | 1.715 |
|  | Sum of precipitation | 0.192 | 1.52 | 0.197% | 0.016% | 3.221 |
|  | Mean perceived temperature | 0.67 | 0.18 | 0.00378% | -0.034% | 1.002 |
|  | Maximum perceived temperature | 0.711 | 0.16 | 0.0063% | -0.034% | 1.055 |
| Spring | Mean ambient temperature | 0.0465 | 2.26 | 0.171% | 0.081% | 2.083 |
|  | Maximum ambient temperature | 0.0534 | 2.5 | 0.19% | 0.084% | 2.457 |
|  | Mean relative humidity | 0.838 | 0.05 | 0.000953% | -0.036% | 1.004 |
|  | Mean windspeed | 0.0955 | 2.29 | 0.124% | 0.051% | 1.963 |
|  | Global radiation | 0.422 | 0.56 | 0.0355% | -0.017% | 1.306 |
|  | Duration of precipitation | 0.821 | 0.07 | 0.00251% | -0.037% | 1.025 |
|  | Sum of precipitation | 0.471 | 0.70 | 0.048% | -0.018% | 1.694 |
|  | Mean perceived temperature | 0.0663 | 2.76 | 0.138% | 0.062% | 1.793 |
|  | Maximum perceived temperature | 0.0738 | 2.24 | 0.175% | 0.071% | 2.461 |

Seasonally stratified univariable GAM analyses results. Presenting (*) the P-value of the smooth function, (**) the F-statistic of the smooth function, (***) the percentage of explained deviance by the model, (****) the percentage of explained variance by the model and (*****) the effective degrees of freedom of the smooth function for the resulted associations. Significant results are highlighted in bold.

Supplementary Table 6 – Results of multivariable GAM analyses

| **Season(s)** | **Meteorological variable** | **GAM P-value^*^** | **GAM F-statistic^**^** | **Deviance explained^***^** | **Variance explained (%)^****^** | **GAM edf^*****^** |
| --- | --- | --- | --- | --- | --- | --- |
| Comprehensive | Mean daily ambient temperature | 0.00155 | 3.65 | 0.205% | 0.149% | 3.440 |
|  | Daily global radiation | 0.98627 | 0.01 | 0.205% | 0.149% | 1.023 |
|  | Mean daily windspeed | 0.00267 | 10.02 | 0.205% | 0.149% | 1.011 |
|  | Mean daily relative humidity | 0.87318 | 0.01 | 0.205% | 0.149% | 1.009 |
| Summer | Mean daily ambient temperature | 0.00789 | 3.35 | 0.393% | 0.229% | 3.346 |
|  | Mean daily windspeed | 0.06398 | 3.43 | 0.393% | 0.229% | 1.004 |
| Fall | Mean daily ambient temperature | 0.0185 | 3.39 | 0.447% | 0.299% | 2.322 |
|  | Mean daily windspeed | 0.006 | 7.54 | 0.447% | 0.299% | 1.003 |
|  | Mean daily relative humidity | 0.5697 | 0.72 | 0.447% | 0.299% | 2.456 |
| Winter | Daily sum of precipitation | 0.281 | 1.27 | 0.239% | 0.085% | 3.249 |
|  | Daily mean windspeed | 0.166 | 1.92 | 0.239% | 0.085% | 1.003 |
| Spring | Mean daily ambient temperature | 0.019 | 3.41 | 0.342% | 0.212% | 2.092 |
|  | Mean daily windspeed | 0.038 | 3.05 | 0.342% | 0.212% | 2.111 |

Multivariable GAM analyses results for all data and for seasonally stratified data. (*) the P-value of the smooth function, (**) the F-statistic of the smooth function, (***) the percentage of explained deviance by the model, (****) the percentage of explained variance by the model and (*****) the effective degrees of freedom of the smooth function for the resulted associations.

Supplementary Table 7 - Results of univariable, comprehensive GAM analyses with lagged daily mean ambient temperature values

| **Lag value (in days)** | **GAM P-value^*^** | **GAM F-statistic^**^** | **Deviance explained (%)^***^** | **Variance explained (%)^****^** | **GAM edf^*****^** |
| --- | --- | --- | --- | --- | --- |
| 0 | 2,5*10^-6^ | 22.04 | 0,113% | 0,096% | 1,015 |
| 1 | 2,47*10^-5^ | 17.29 | 0,0909% | 0,075% | 1,028 |
| 2 | 1,07*10^-5^ | 18.82 | 0,0995% | 0,085% | 1,026 |
| 3 | 2,74*10^-6^ | 20.73 | 0,118% | 0,102% | 1,057 |
| 4 | 1,24*10^-5^ | 15.2 | 0,116% | 0,099% | 1,227 |
| 5 | 4,57*10^-5^ | 6.20 | 0,139% | 0,104% | 3,179 |
| 6 | 1,53*10^-6^ | 6.86 | 0,191% | 0,146% | 4,167 |
| 7 | 7,18*10^-6^ | 10.67 | 0,139% | 0,114% | 1,992 |

(*) the P-value of the smooth function, (**) the F-statistic of the smooth function (***) the percentage of explained deviance by the model, (****) the percentage of explained variance by the model and (*****) the effective degrees of freedom of the smooth function for the resulted associations.

Supplementary figure 1 – Assignment of weather stations to nearby psychiatric institutions


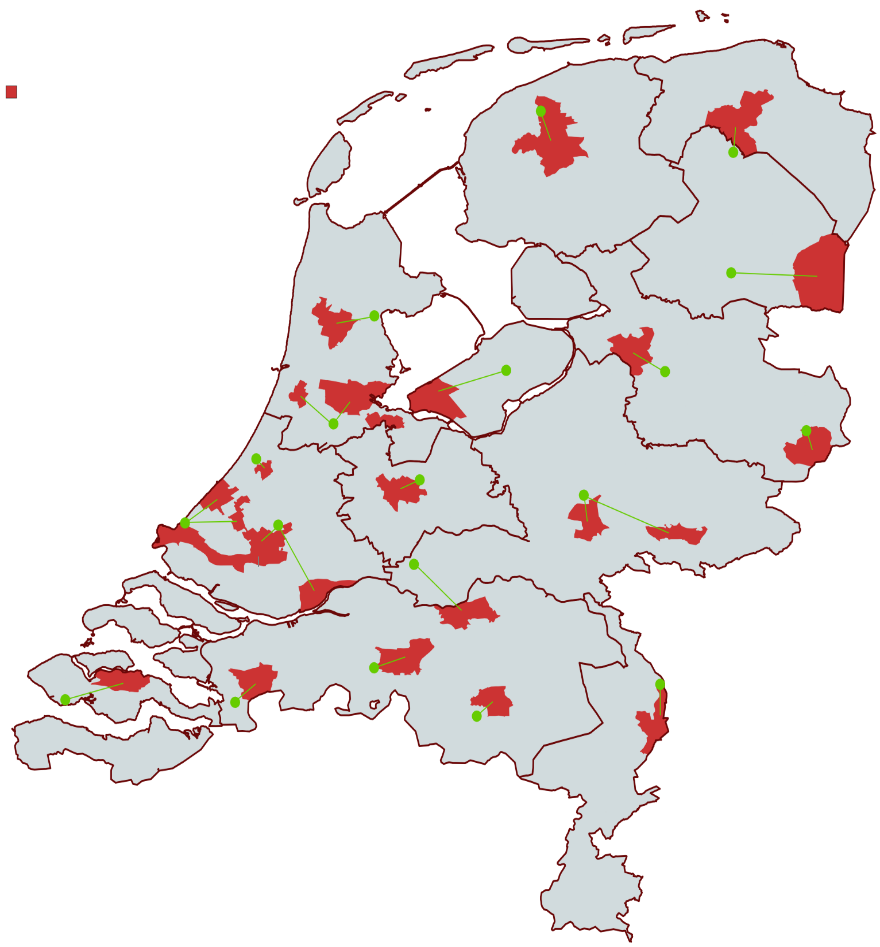


= most populous city for each psychiatric institute

= weather stations

Supplementary Figure 2 – Diagnostic plots of the comprehensive ‘mean daily ambient temperature’ GAM analysis: (A) QQ-plot and (B) histogram of residuals

| 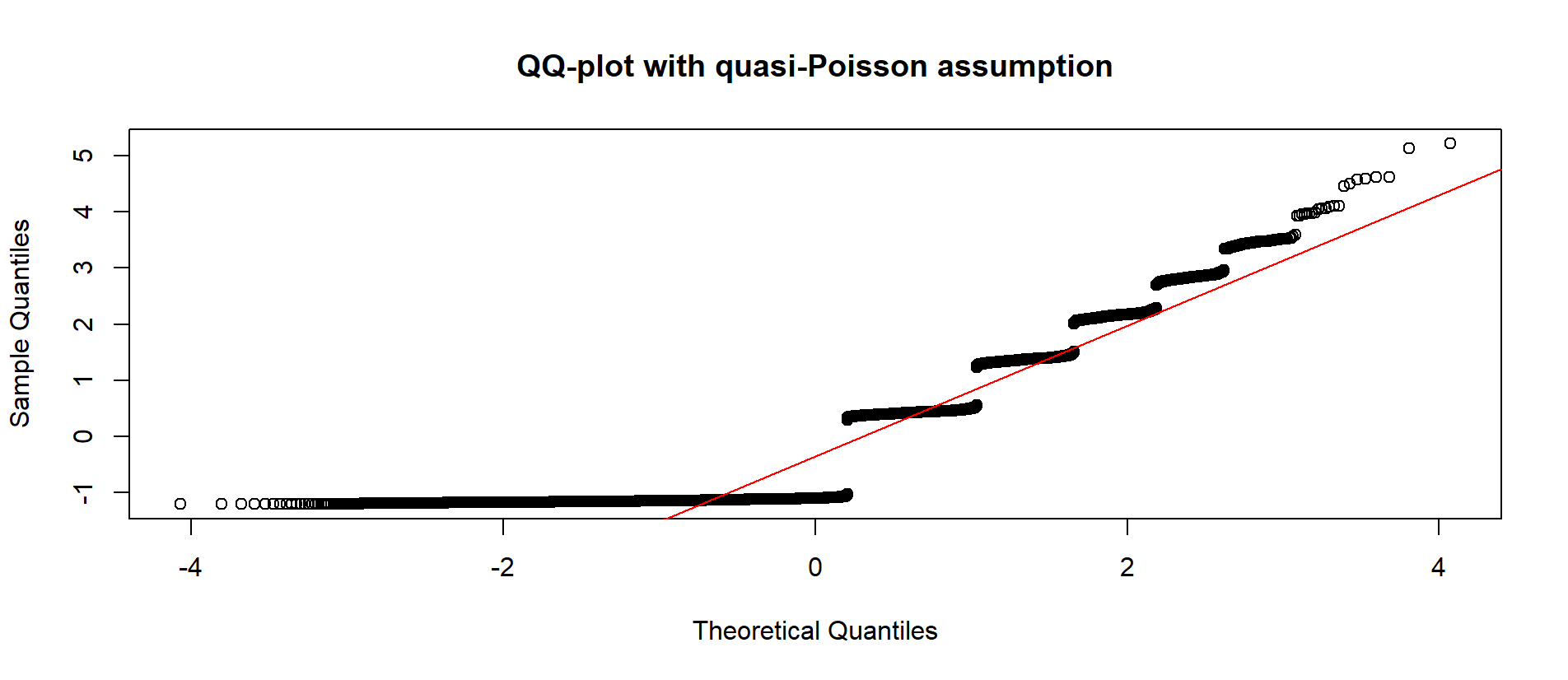  **B**  **A** |
| --- |
| 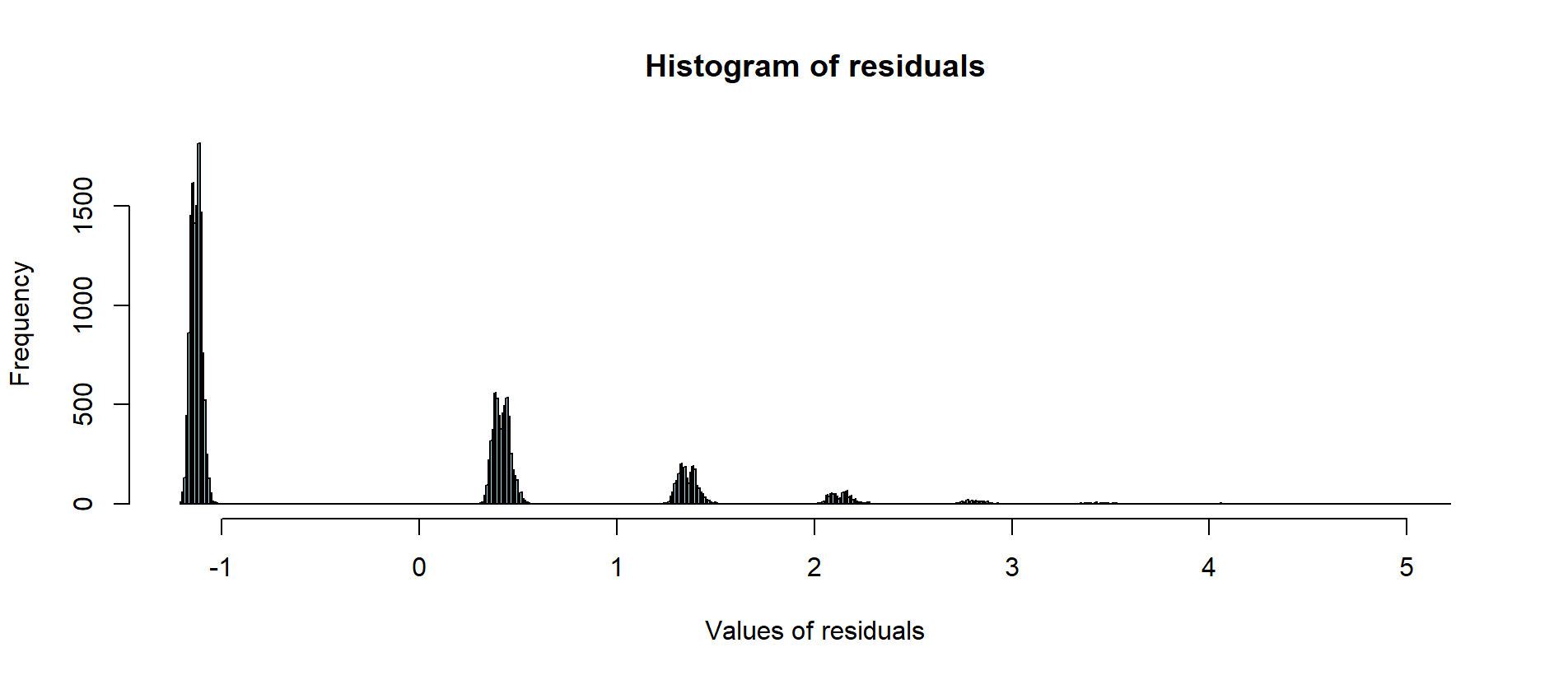 |

Supplementary Figure 3 – Distribution of daily involuntary admission incidence


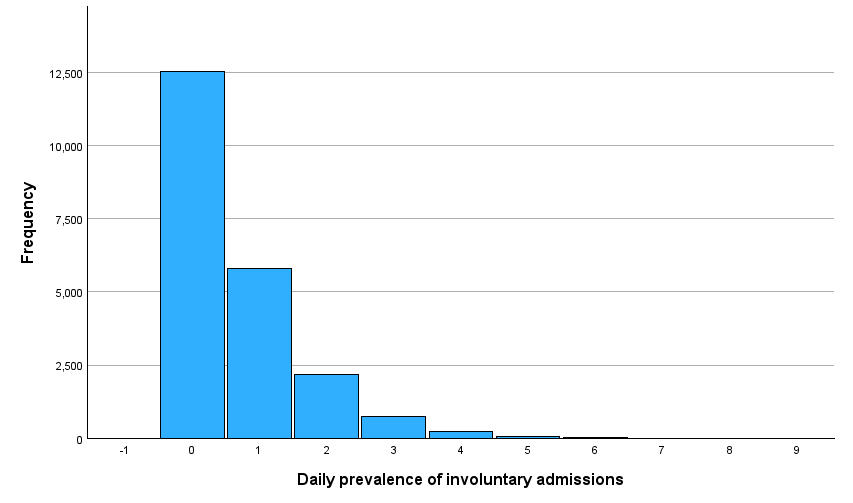


Figure 2. Histogram of the distribution for daily involuntary admissions prevalence.

Supplementary Figure 4 – Distribution graphs for meteorological variables in all data

| 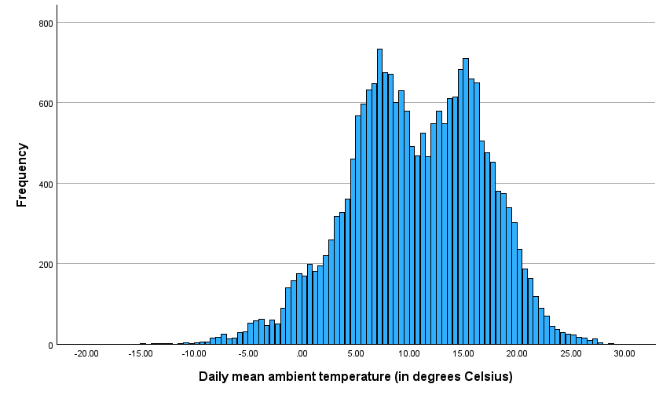  **A** | 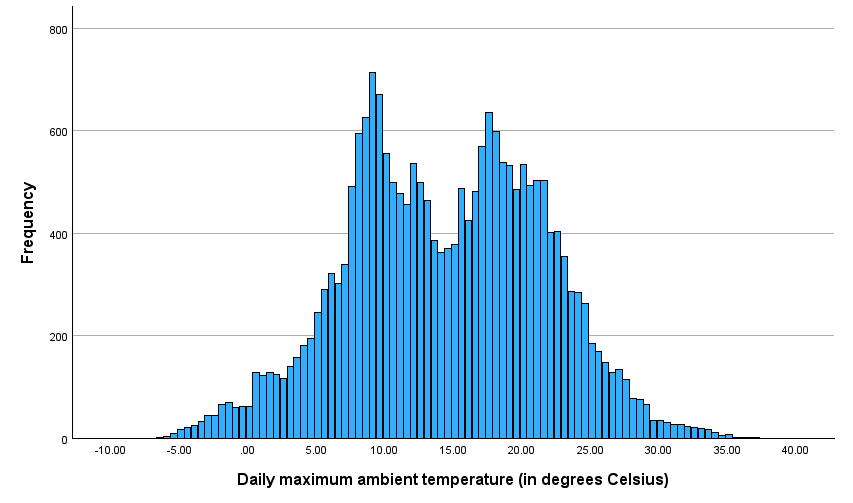  **B** |
| --- | --- |
| 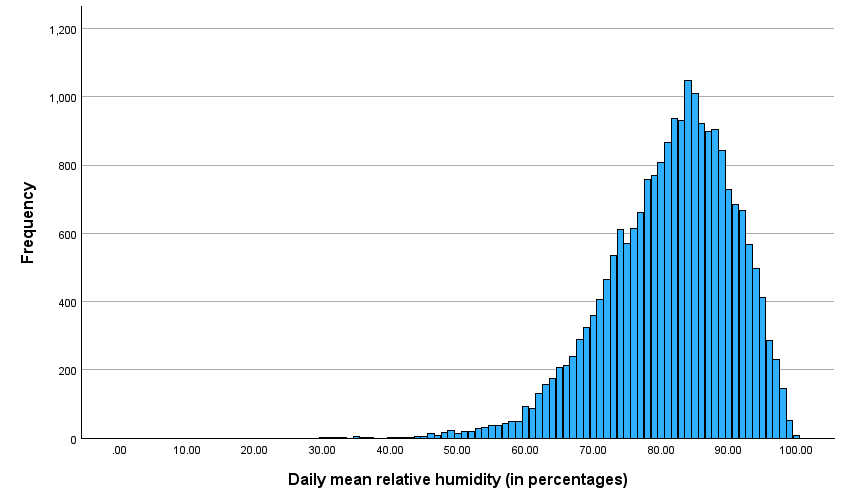  **C** | 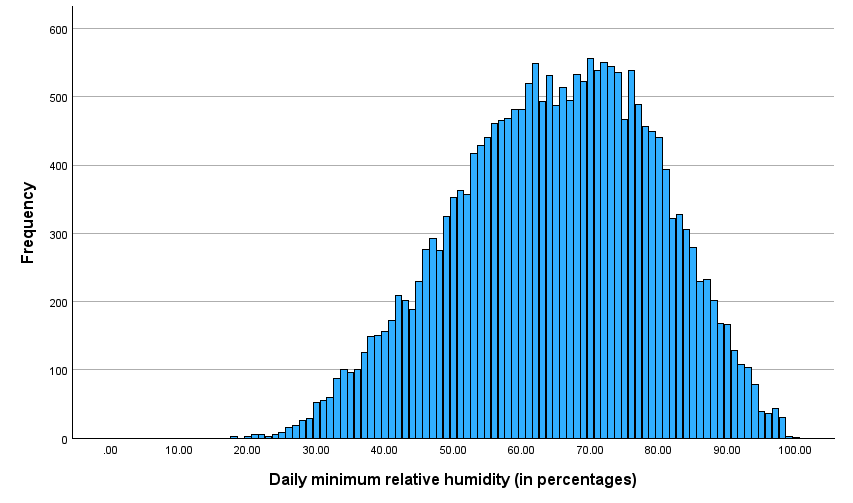  **F**  **D** |
| 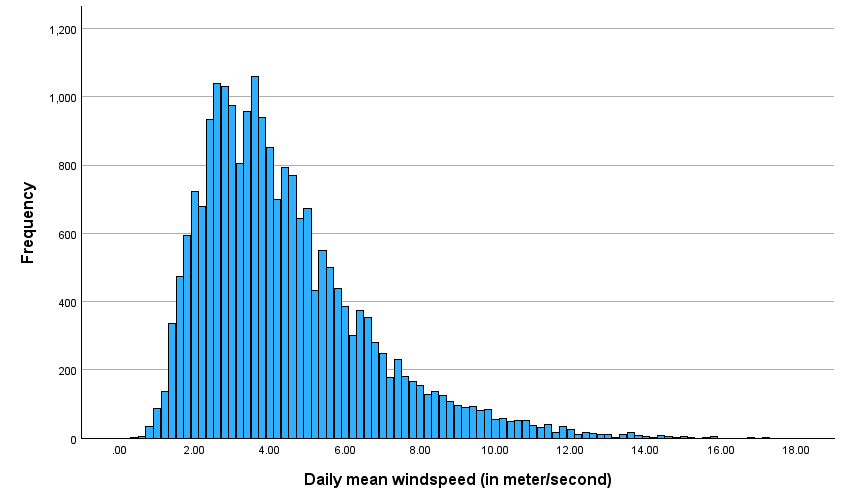  **E** | 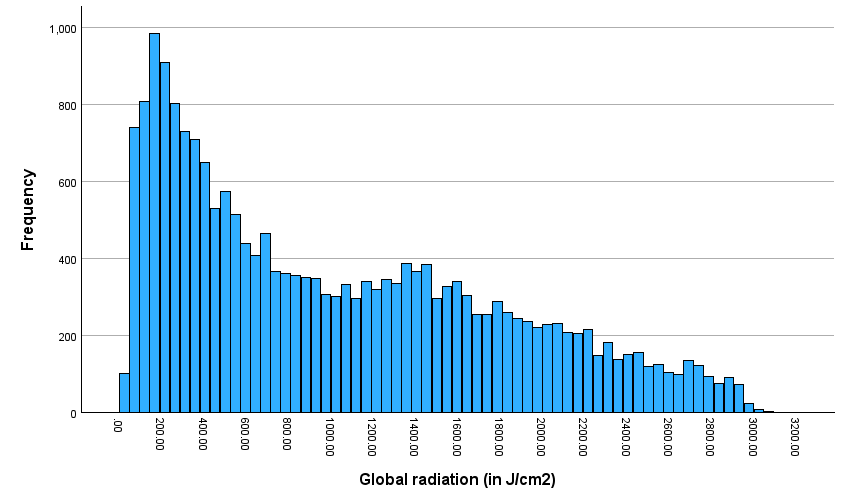 |
| 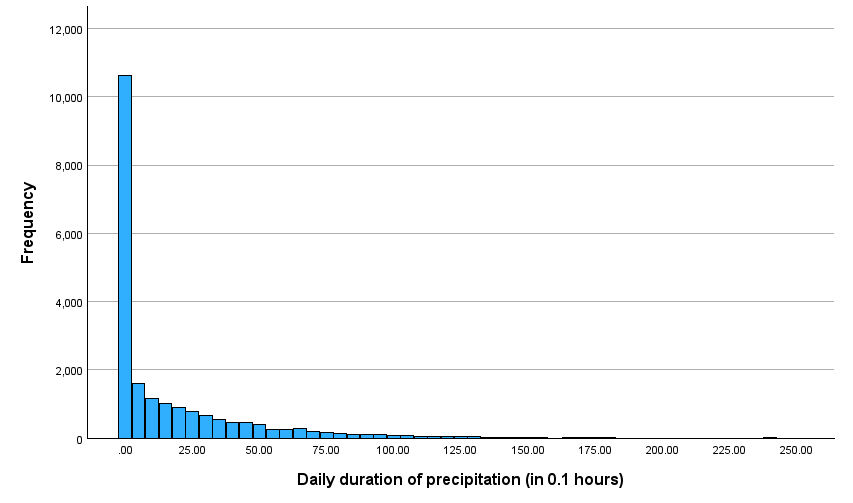  **G** | 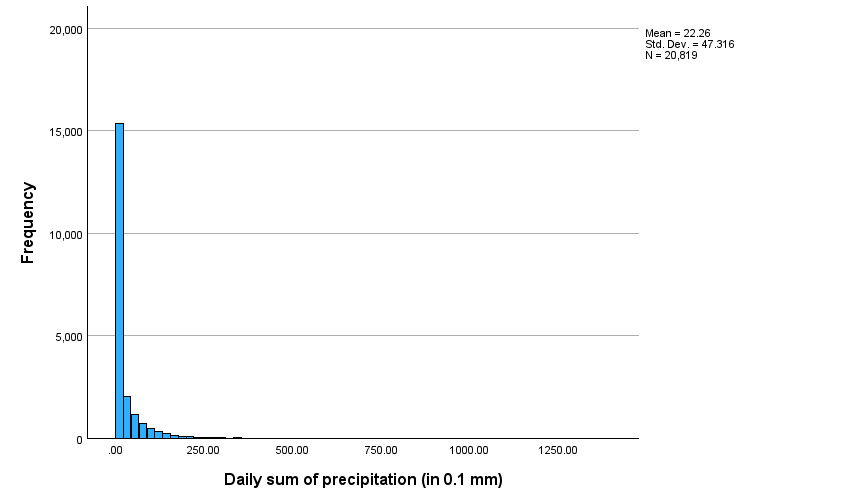  **H** |

Supplementary Figure 2. Distribution of (A) daily mean ambient temperature, (B) daily maximum ambient temperature, (C) daily mean relative humidity, (D) daily minimum relative humidity, (E) daily mean windspeed, (F) global radiation, (G) daily duration of precipitation and (H) daily sum of precipitation.

Supplementary Figure 5 – Distribution graphs of seasonally stratified variables

|  | **Summer** | **Fall** | **Winter** | **Spring** |
| --- | --- | --- | --- | --- |
| **A** | 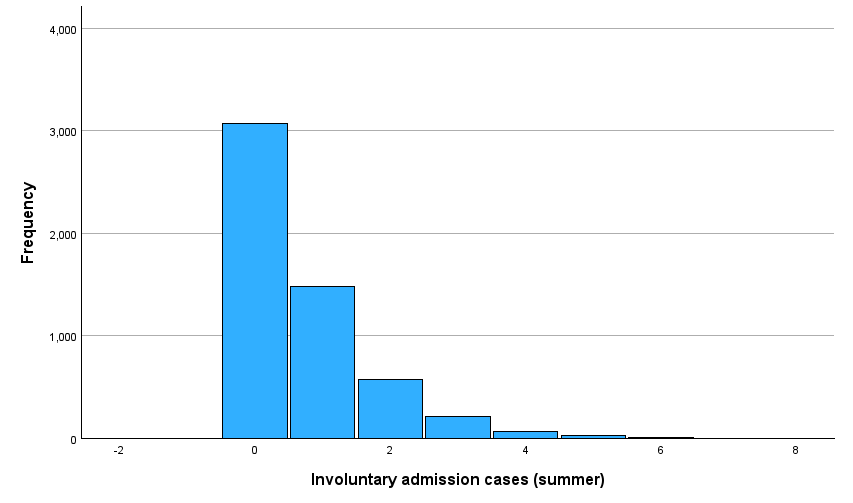 | 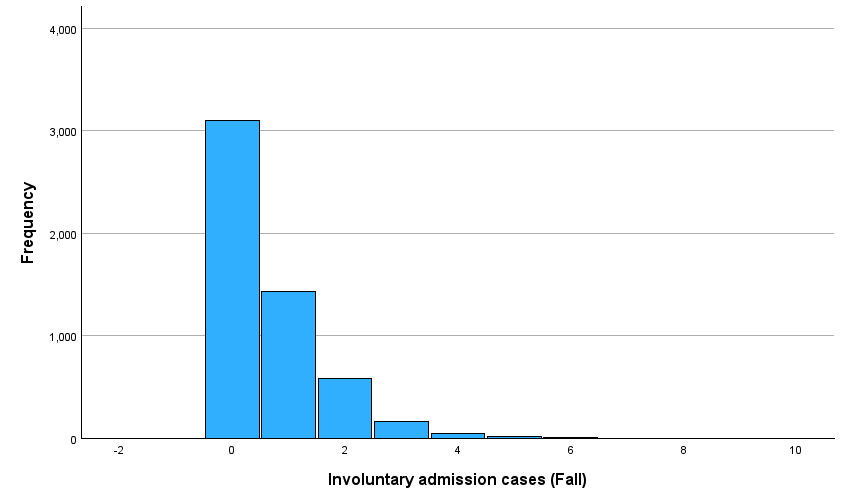 | 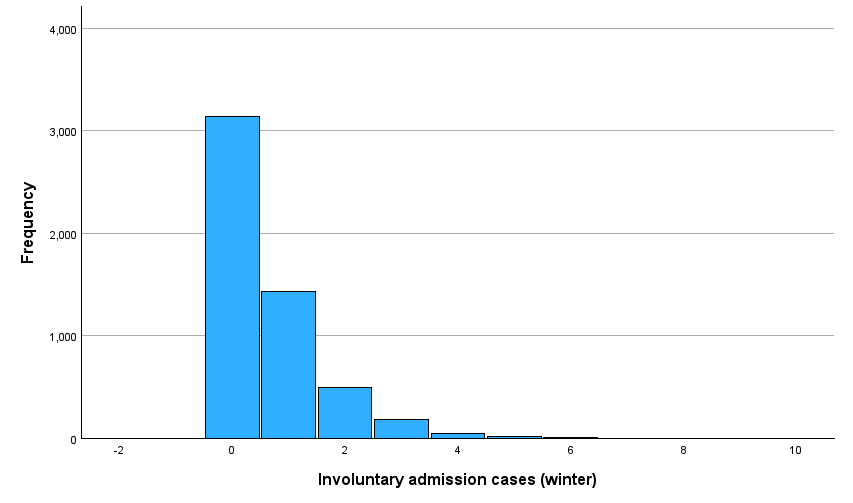 | 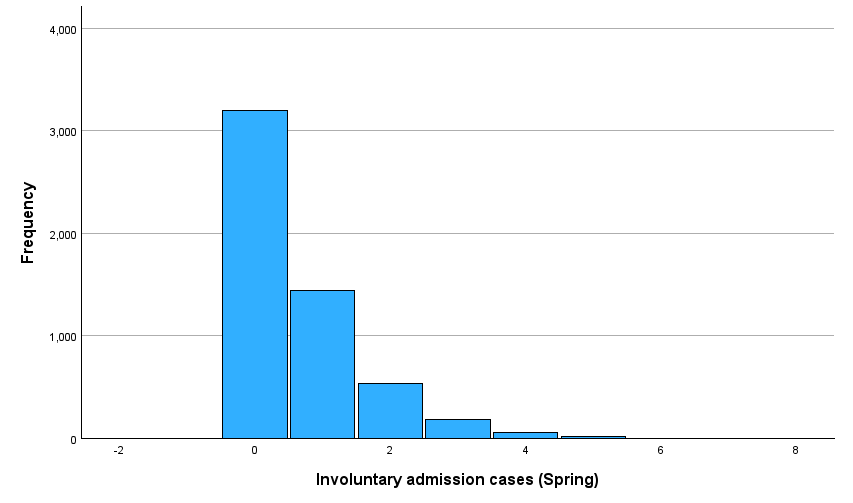 |
| **B** | 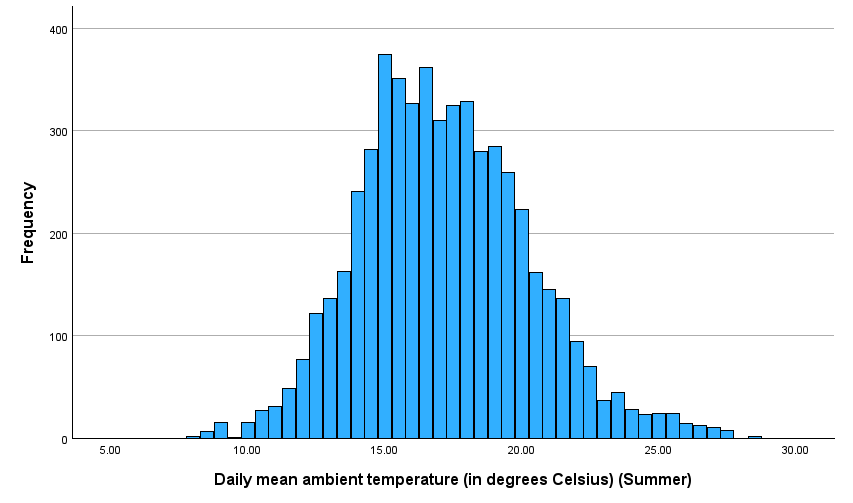 | 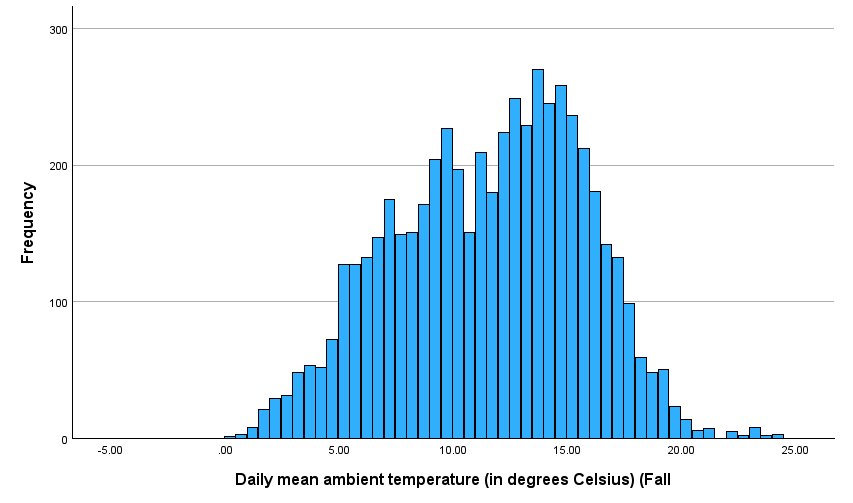 | 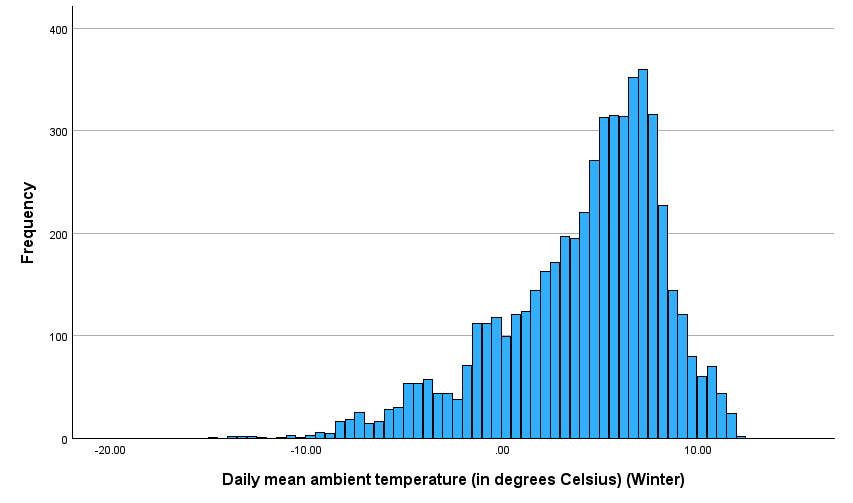 | 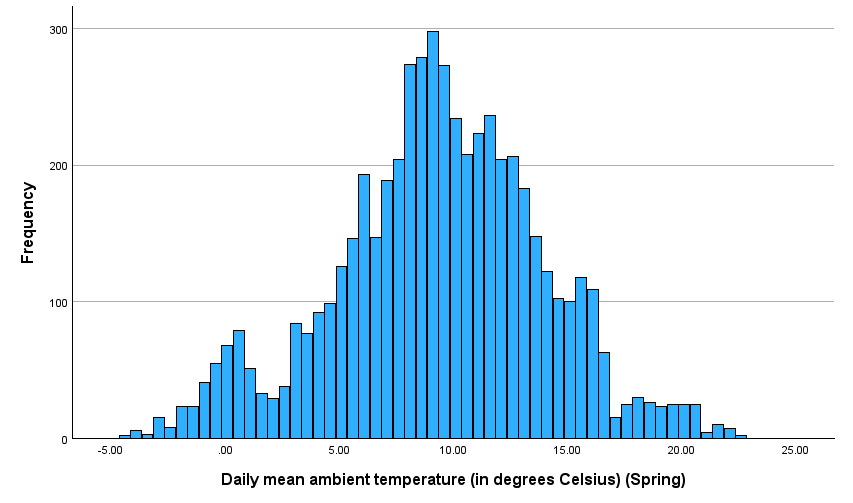 |
| **C** | 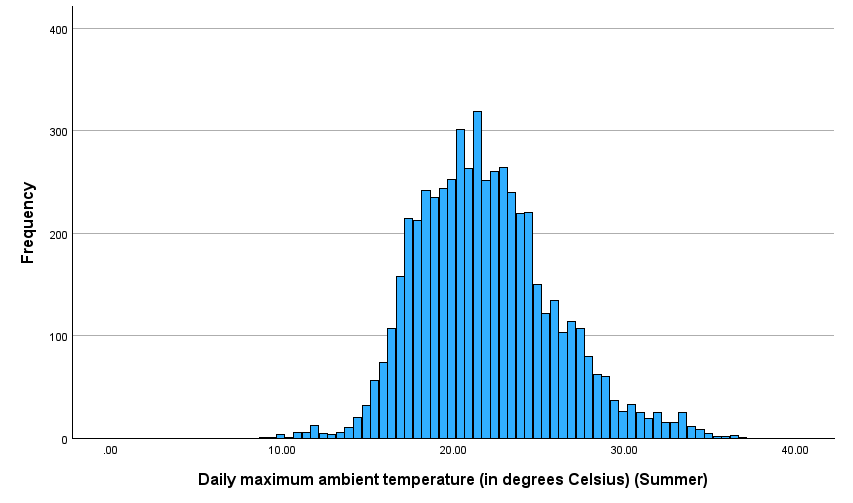 | 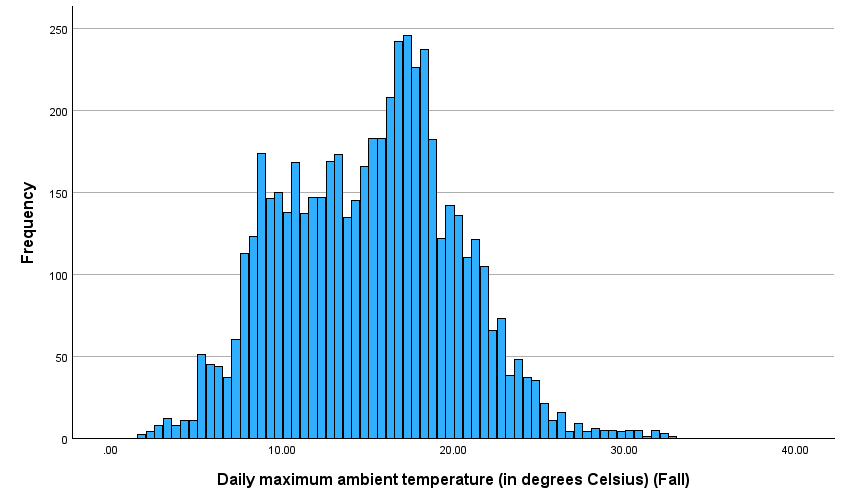 | 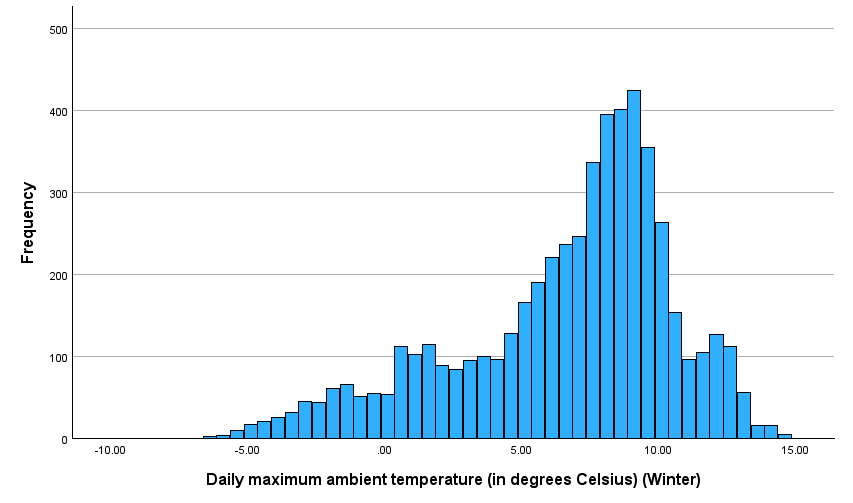 | 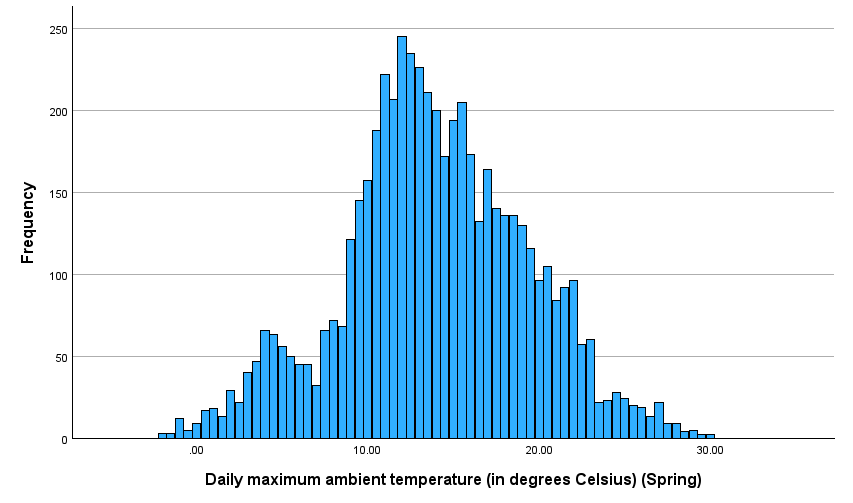 |
| **D** | 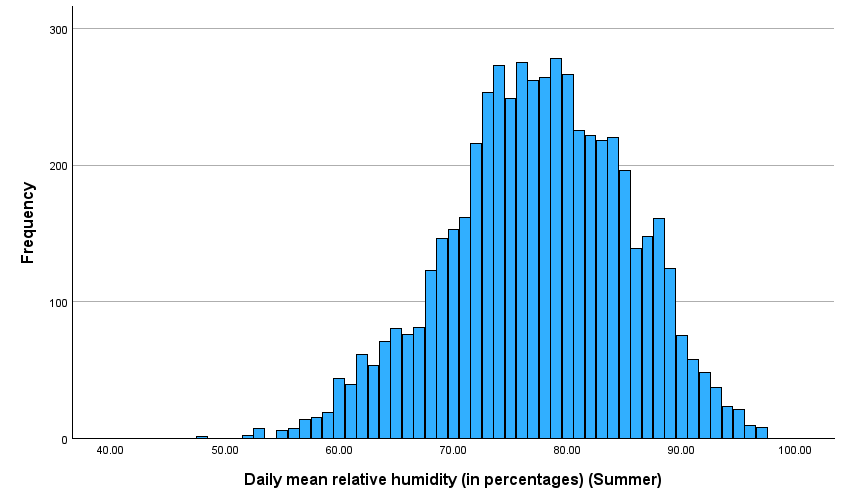 | 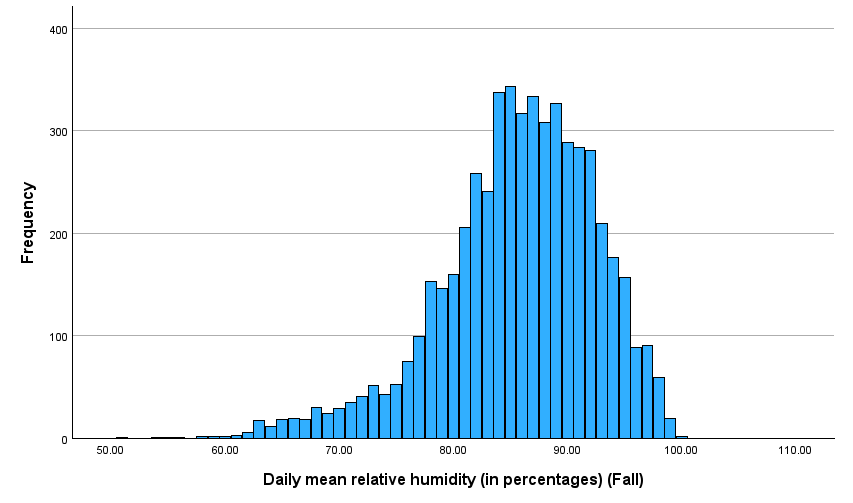 | 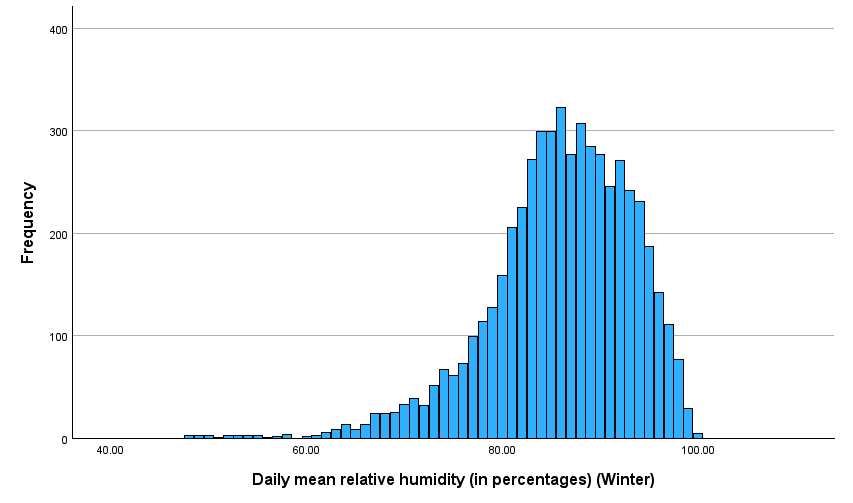 | 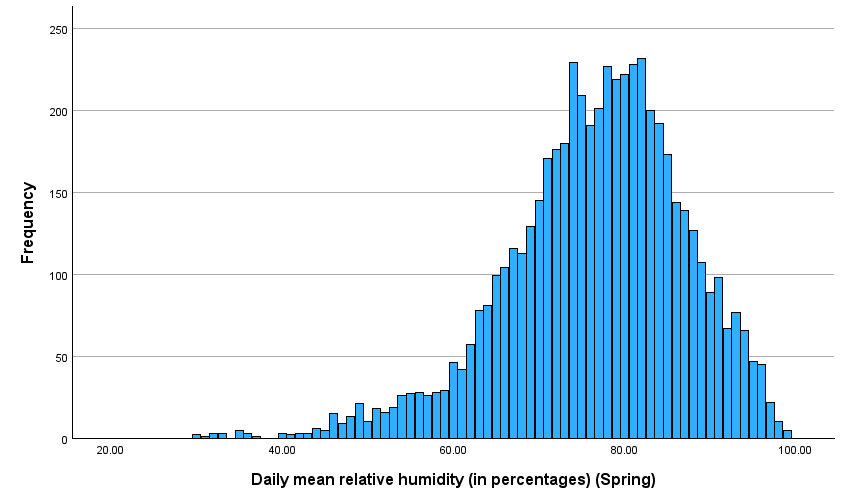 |
| **E** | 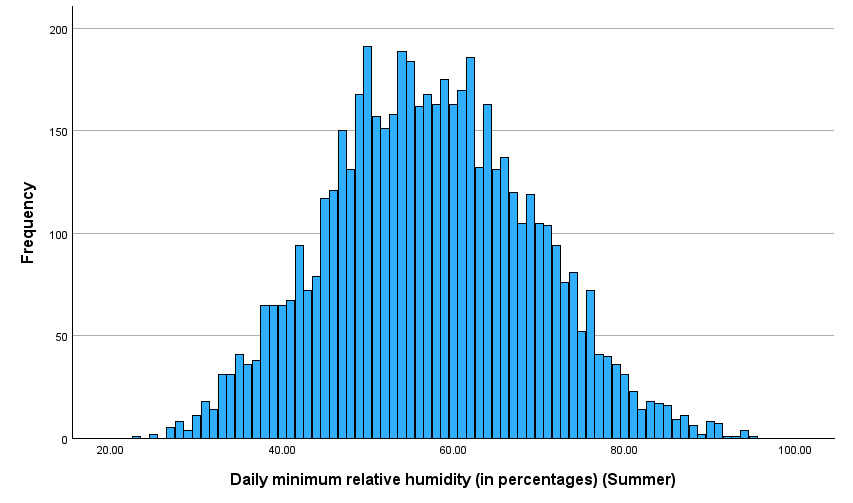 | 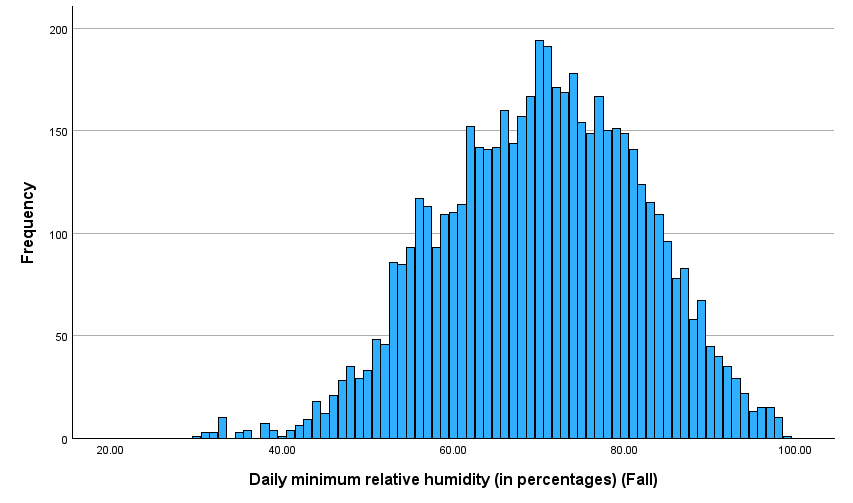 | 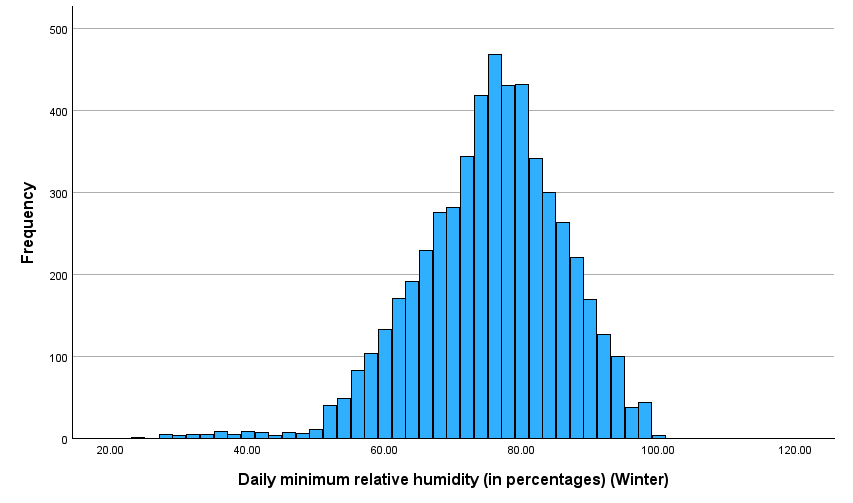 | 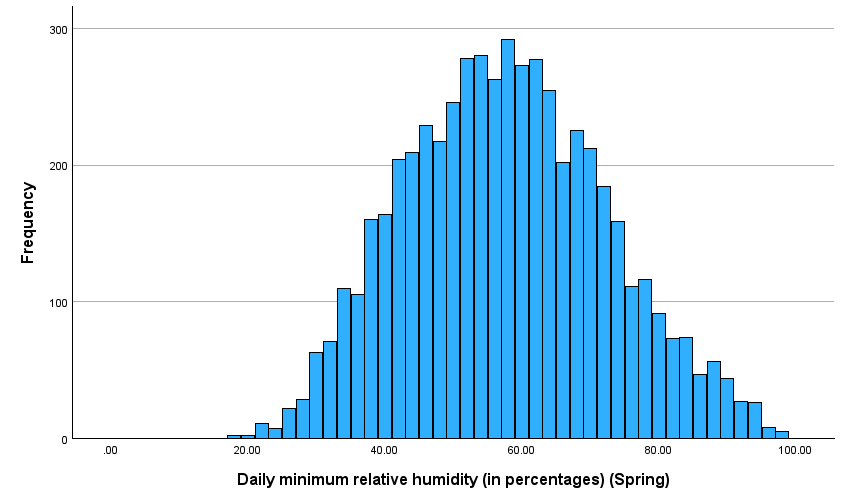 |
| **F** | 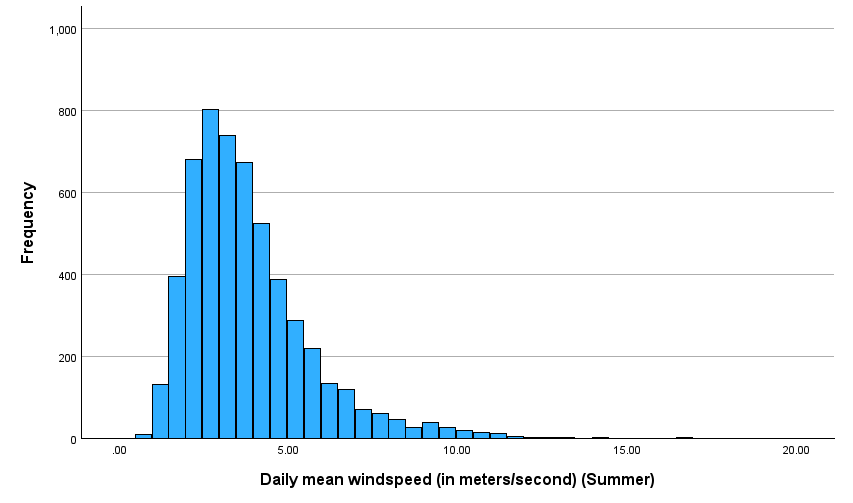 | 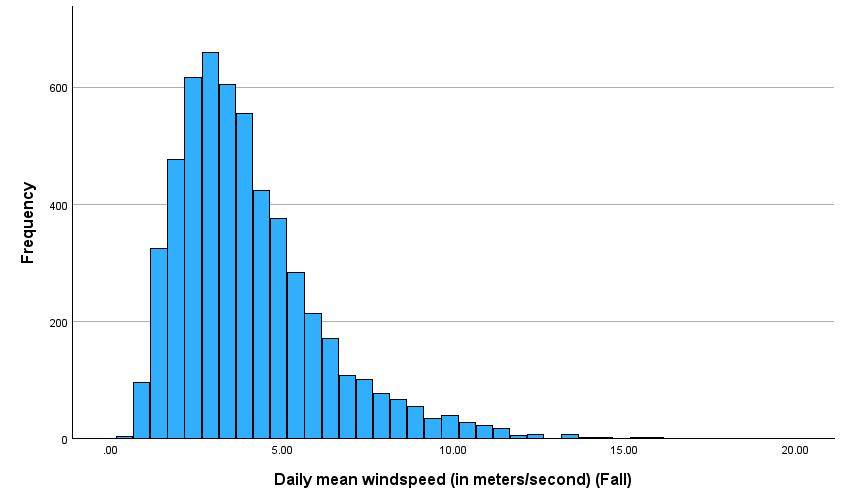 | 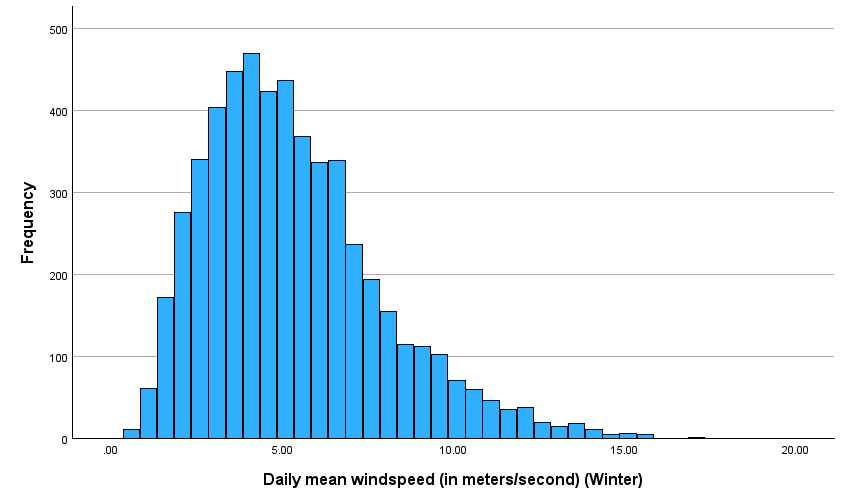 | 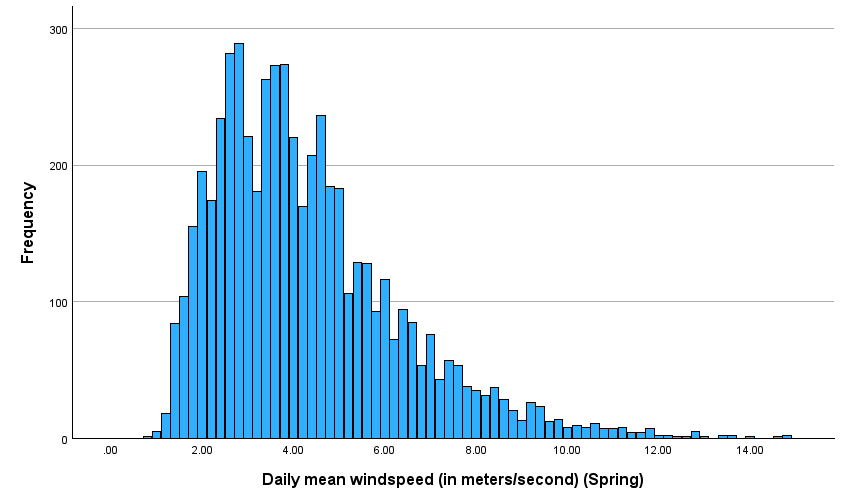 |
| **G** | 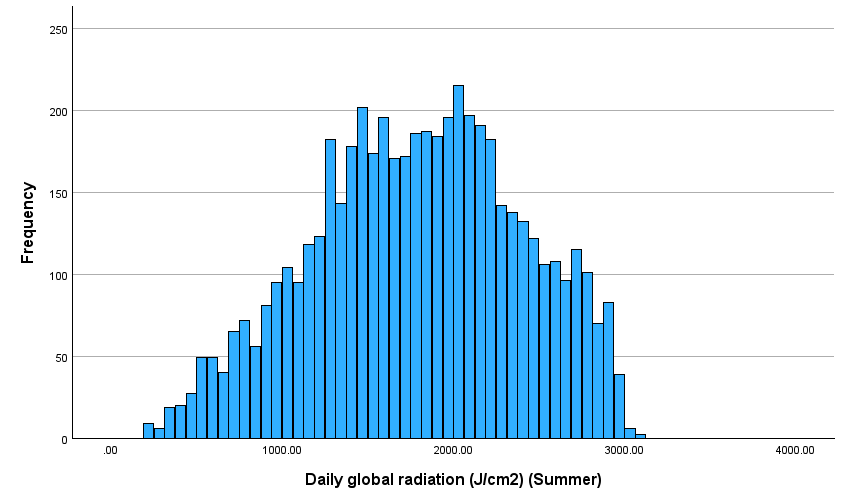 | 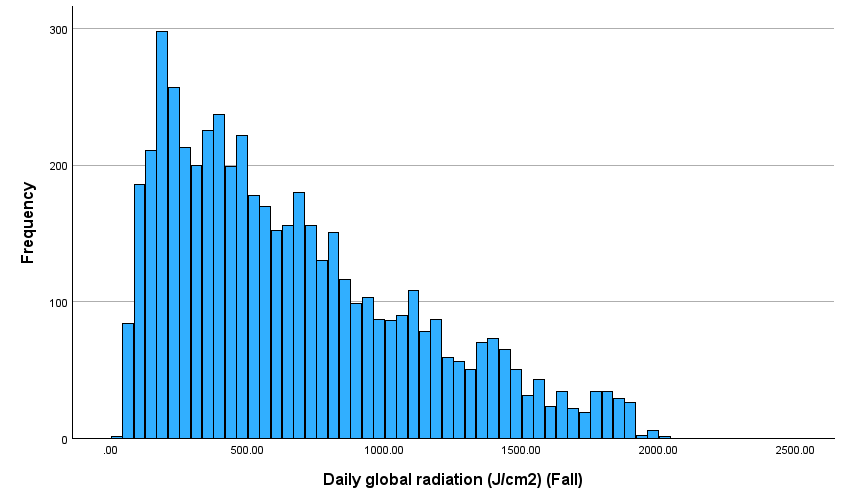 | 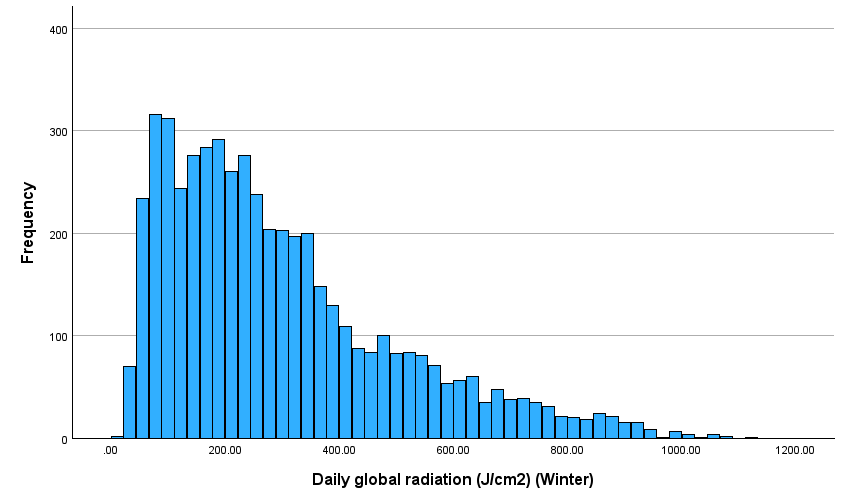 | 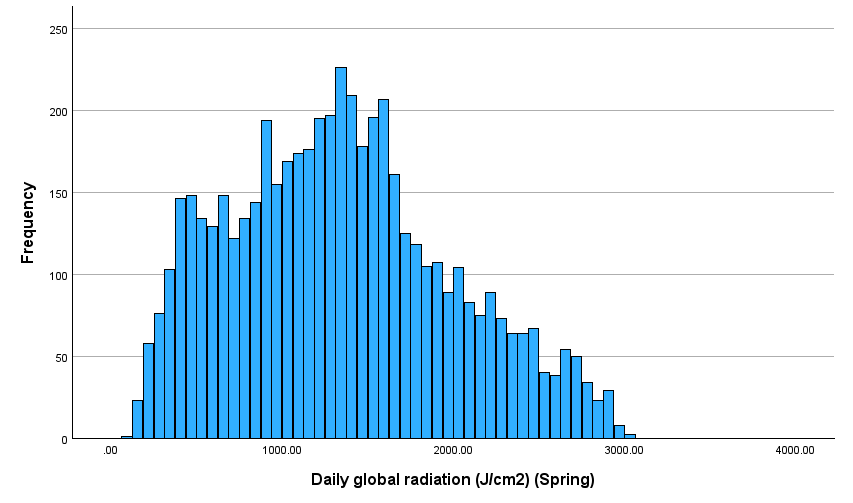 |
| **H** | 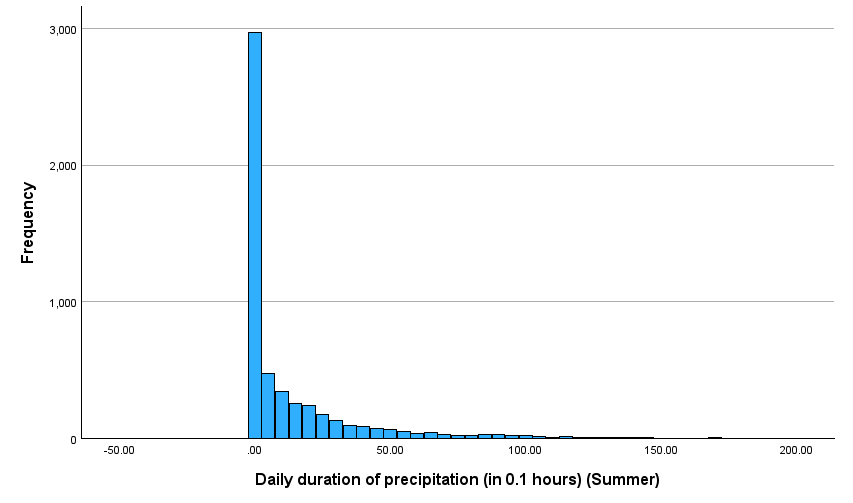 | 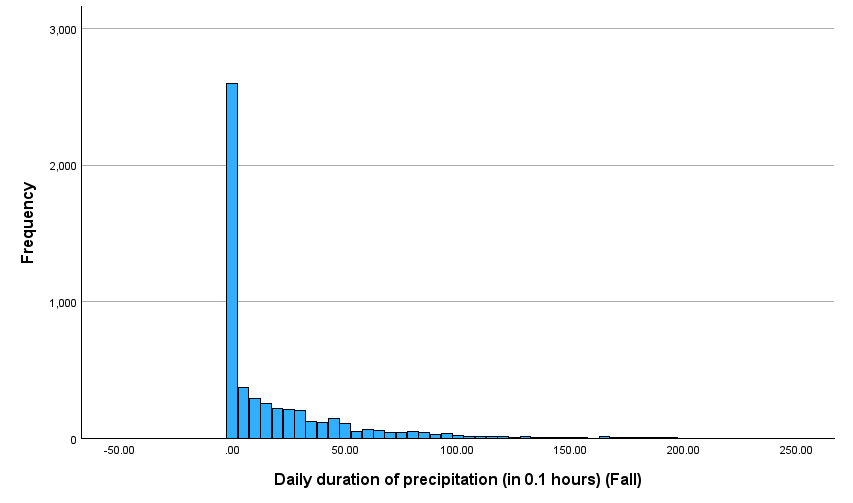 | 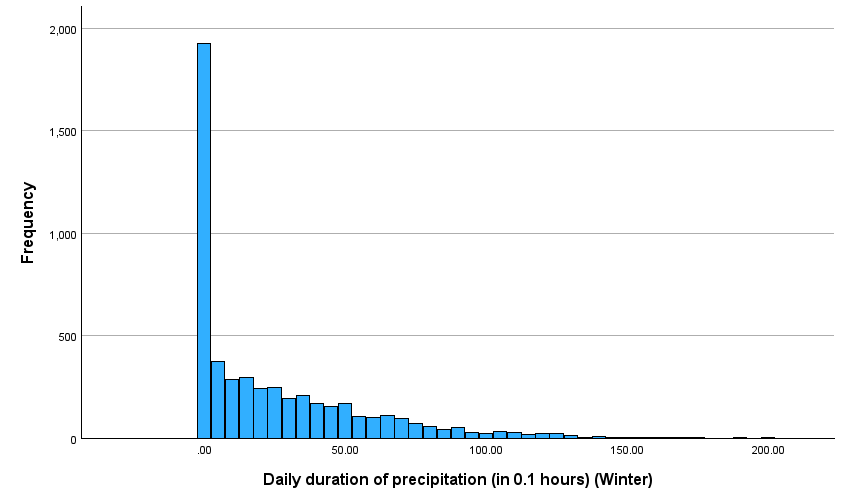 | 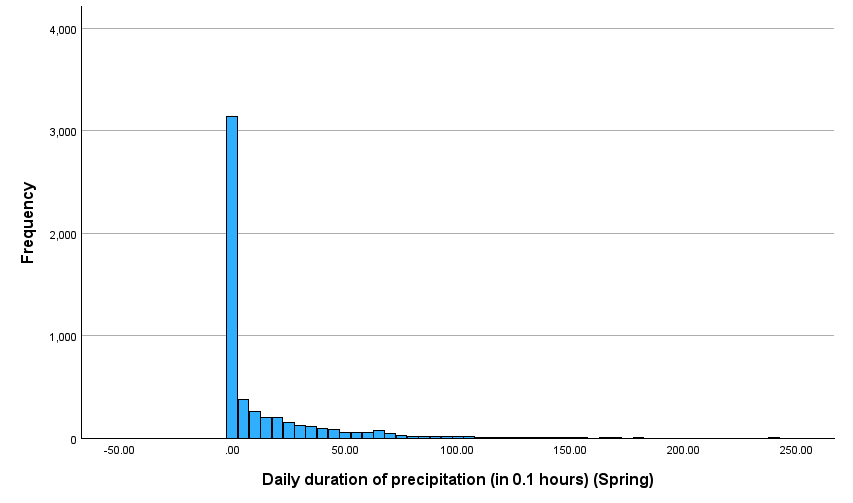 |
| **I** | 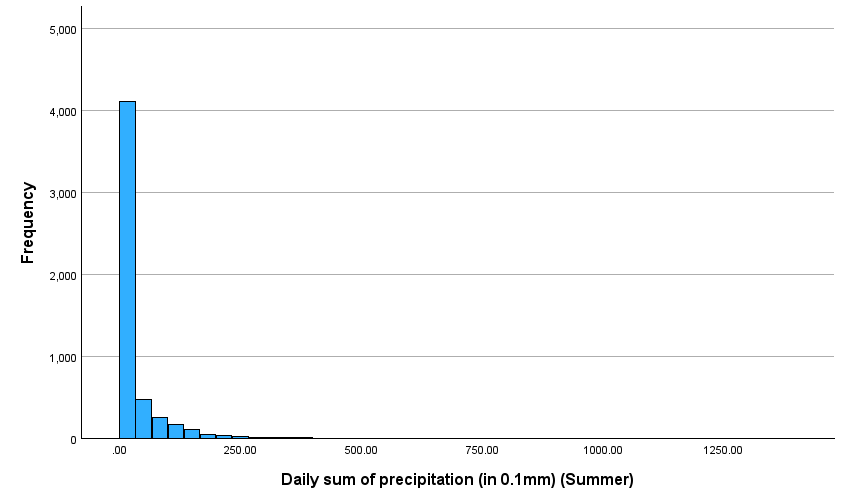 | 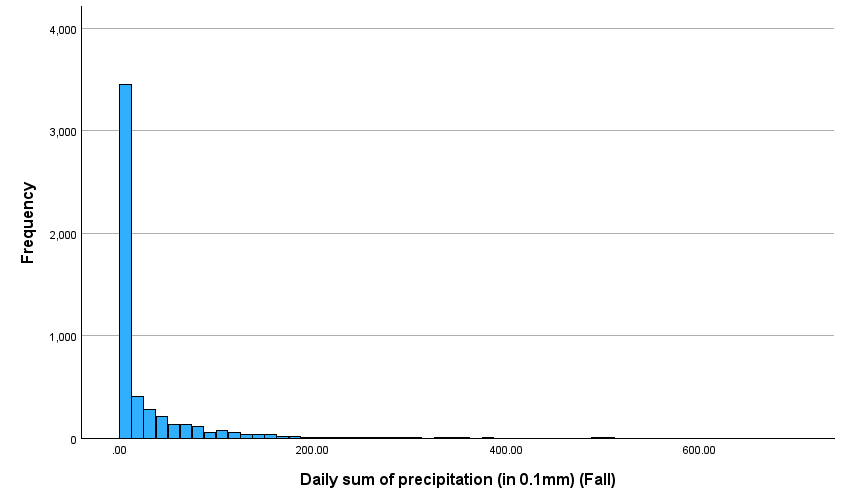 | 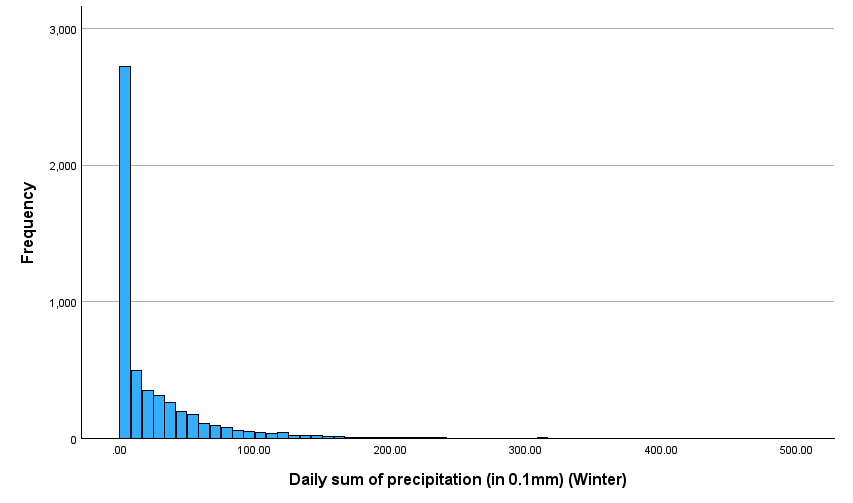 | 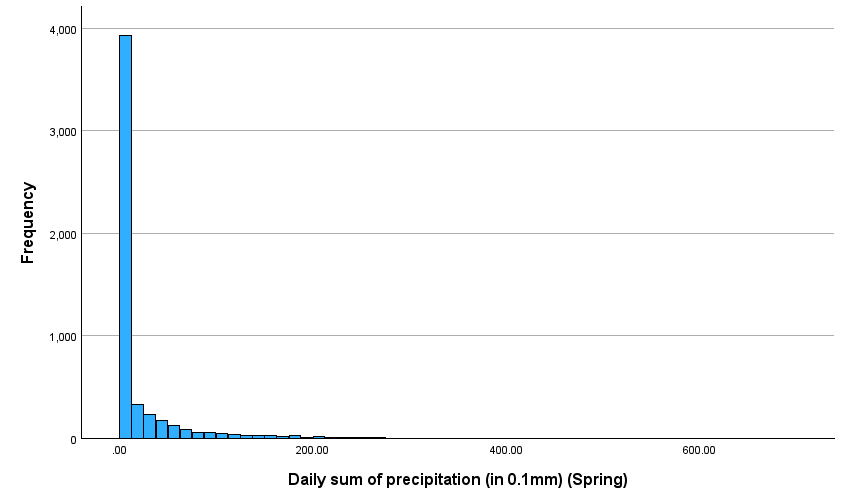 |

*Supplementary Figure 3. Seasonally stratified distribution graphs for (A) involuntary admission cases, (B) Daily mean ambient temperature, (C) Daily maximum ambient temperature, (D) Daily mean relative humidity, (E) Daily minimum relative humidity, (F) Daily mean windspeed, (G) Daily global radiation, (H) Daily duration of precipitation, (I) Daily sum of precipitation.*

| 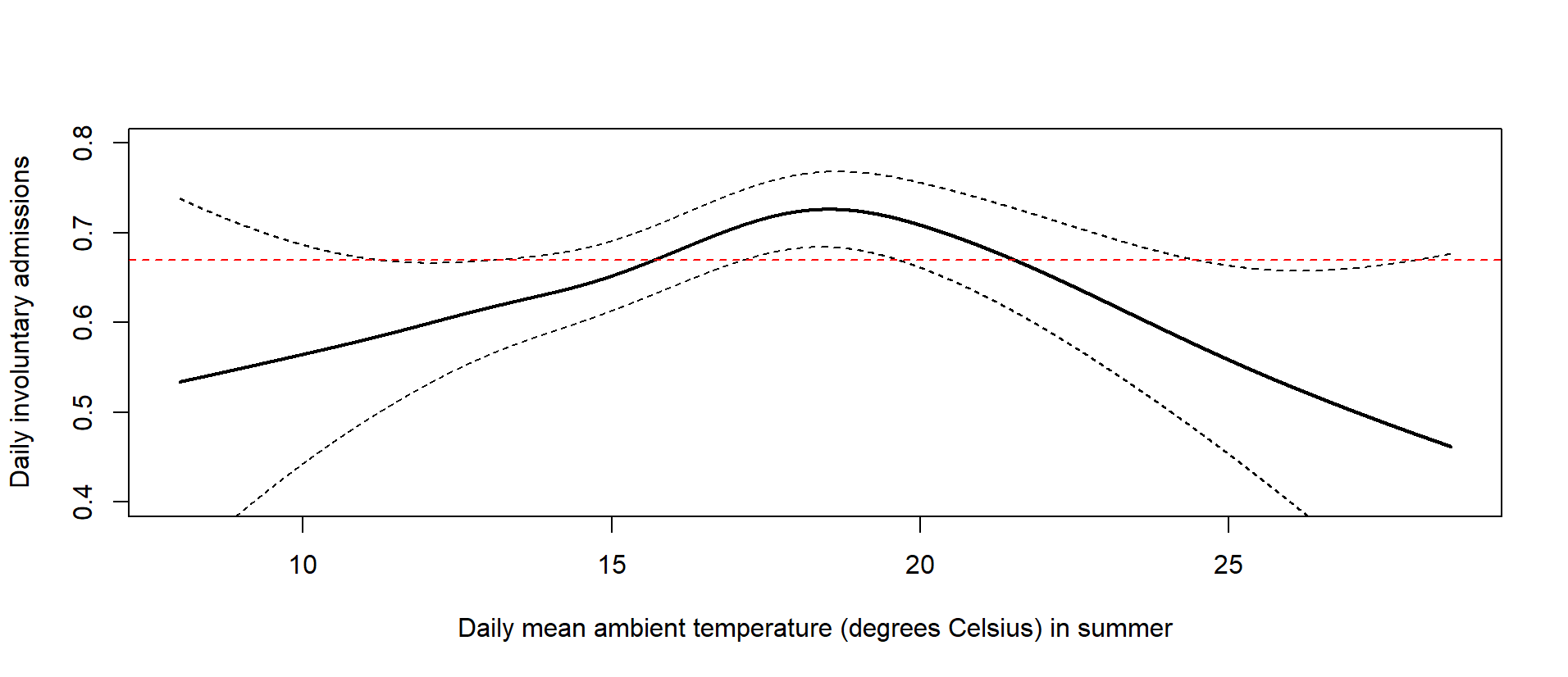  **A** |
| --- |
| 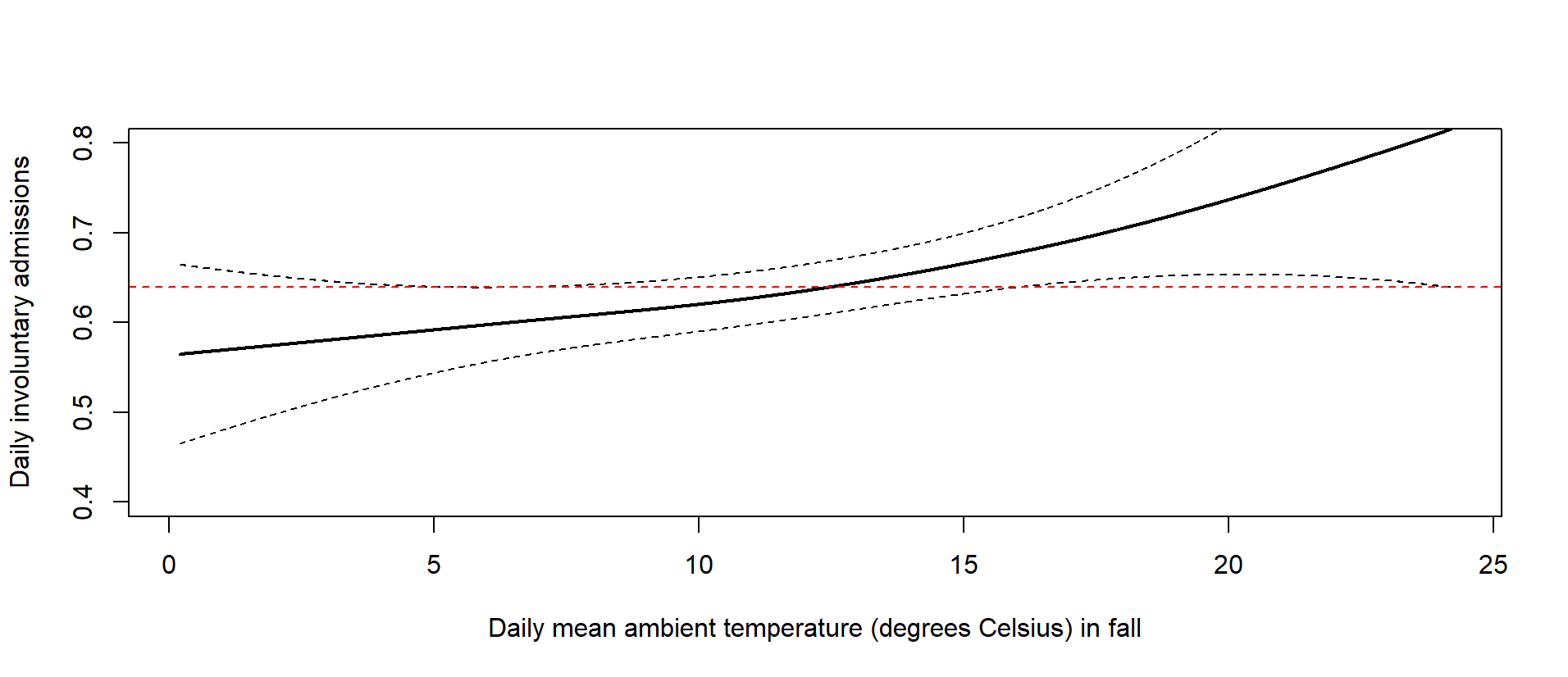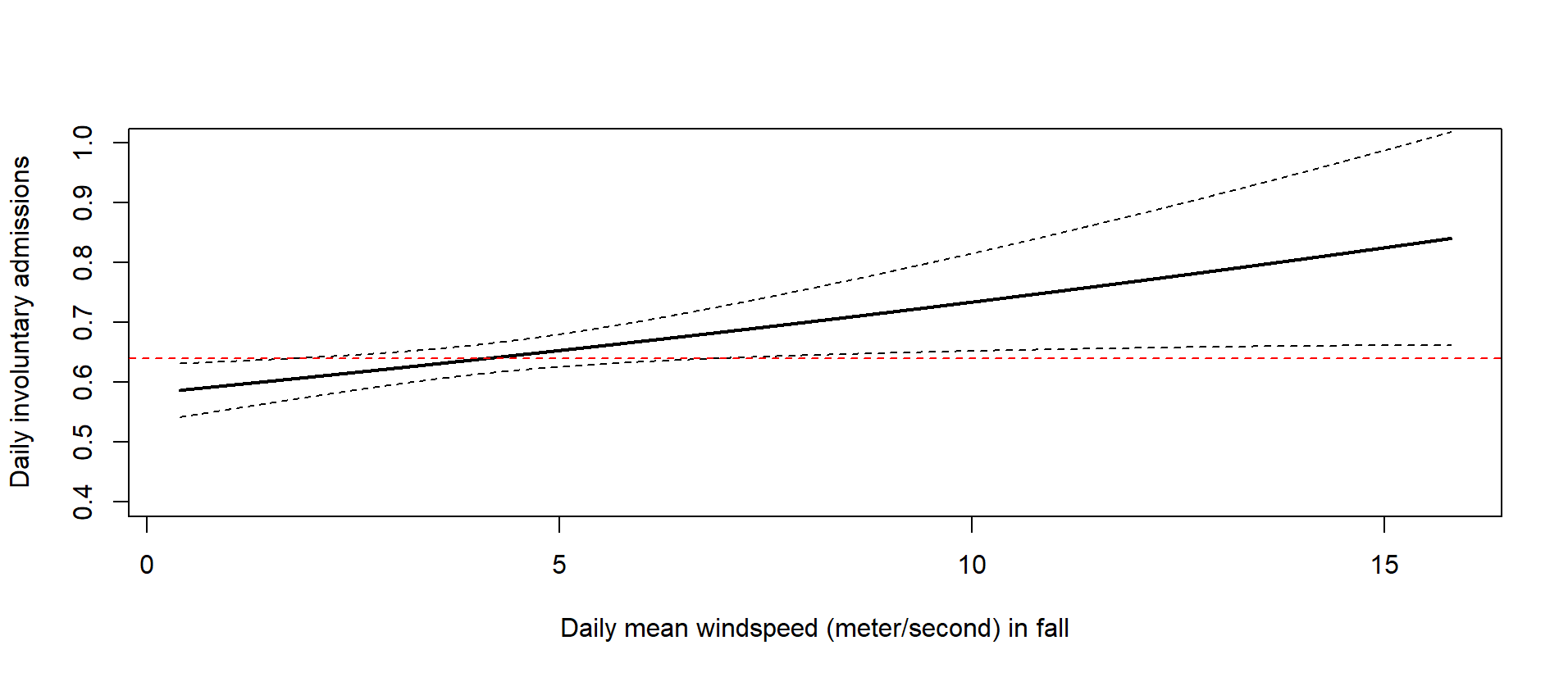  **B** |
| 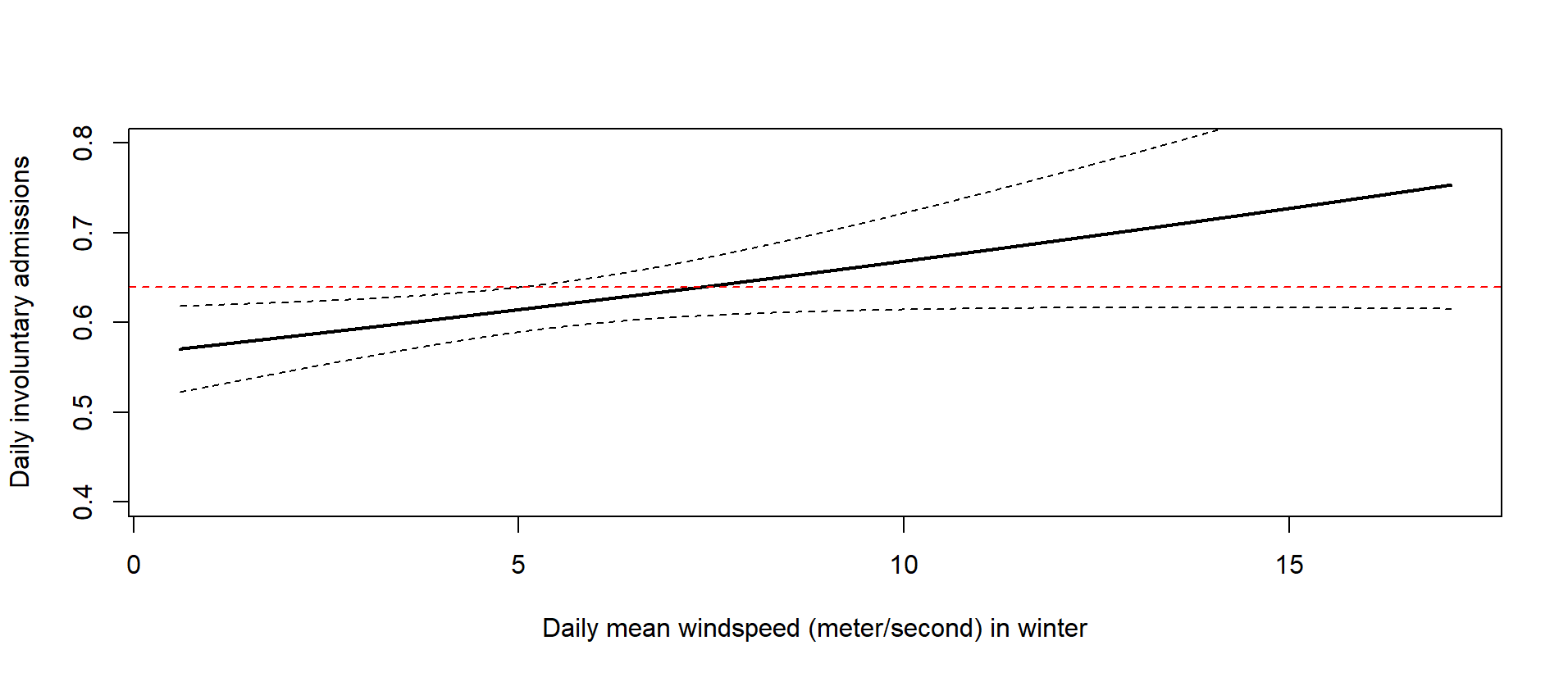  **C** |
| 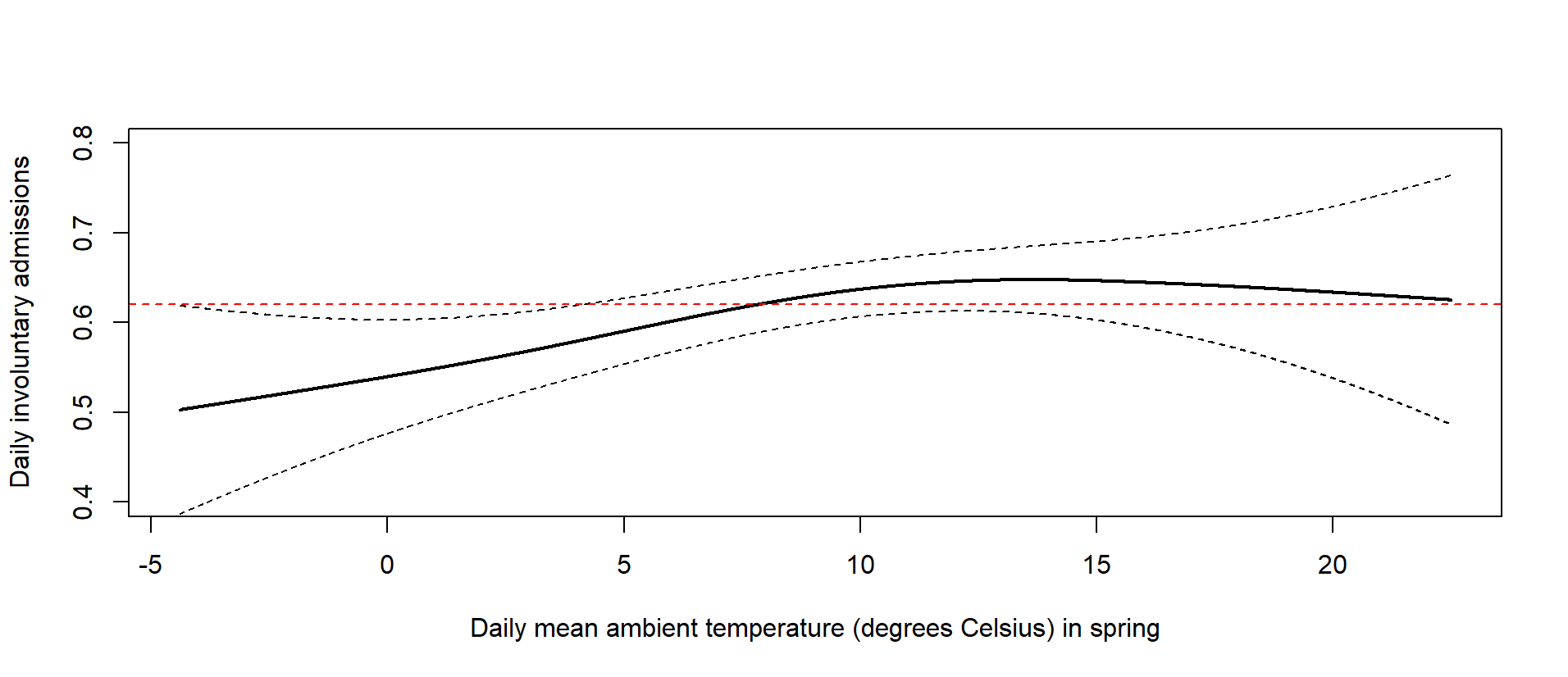  **D** |

Supplementary Figure 6 – Association plots for the most strongly associated meteorological variables in univariable, seasonally stratified GAMs

*(A) summer, (B) fall, (C) winter and (D) spring.* *The y-axes represent the expected daily numbers of involuntary admissions according to the Generalized Additive Models (GAMs) we used, averaged per psychiatric institution, and the x-axes the values of each significantly associated meteorological variable. The dotted black line represents the 95% confidence interval of the association, the dotted red line the mean daily involuntary admissions numbers.*

Supplementary Figure 7 – Association plots of significantly associated meteorological variables in multivariable GAMs.

| 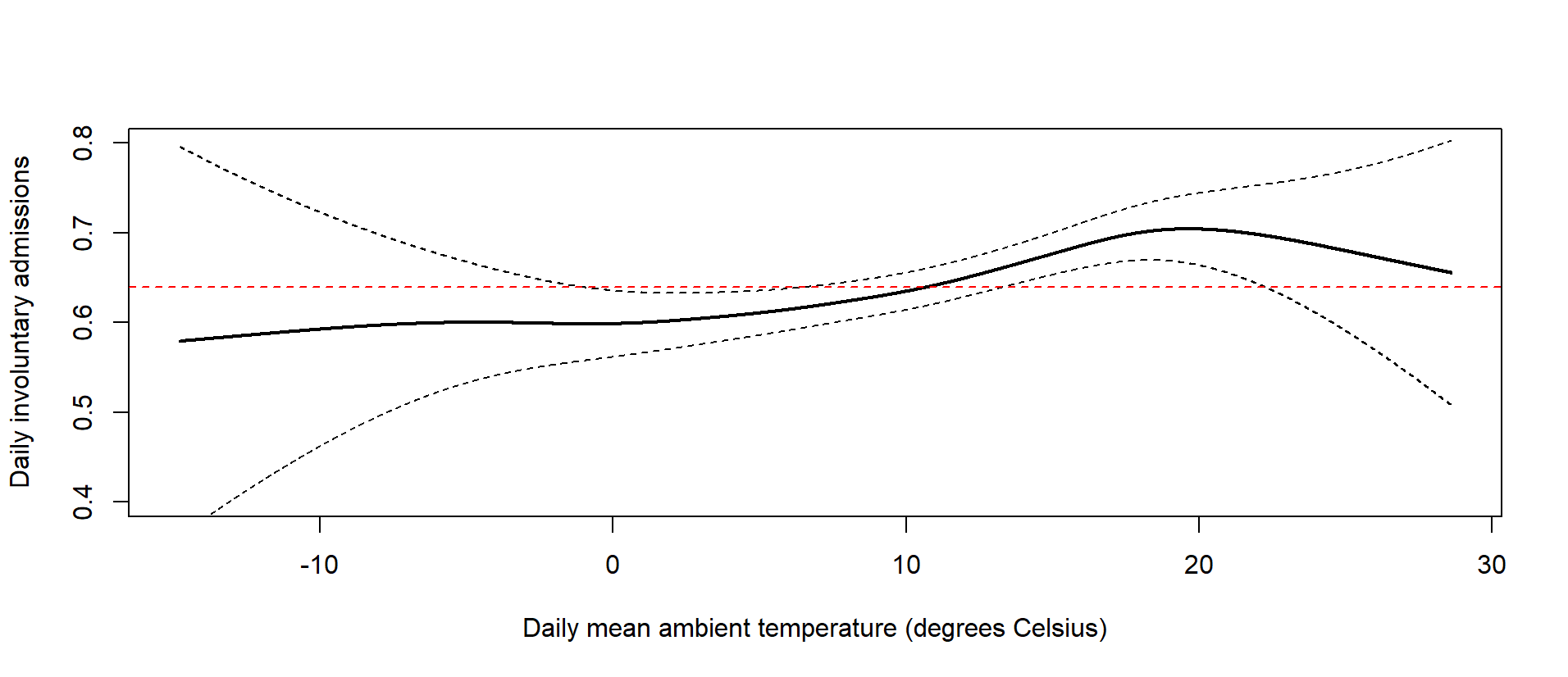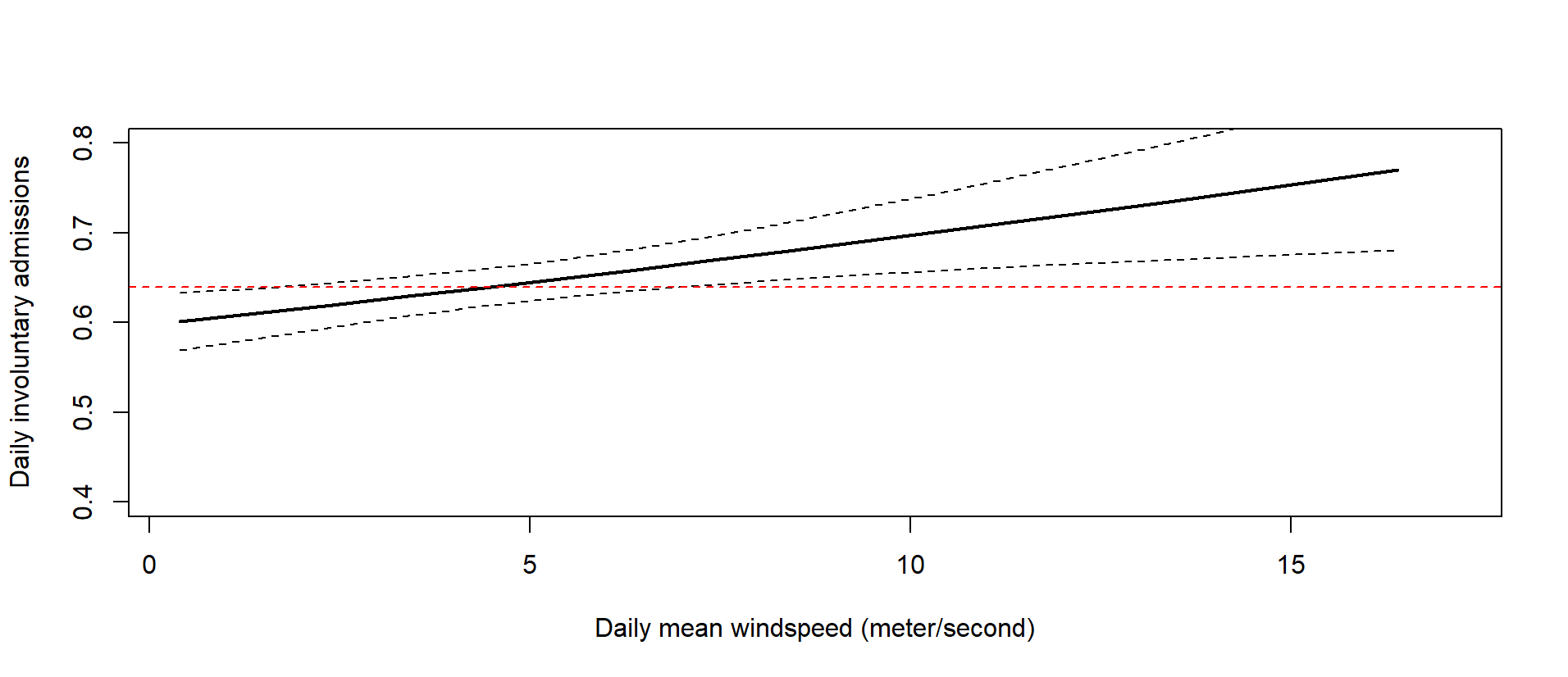  **A** |
| --- |
| 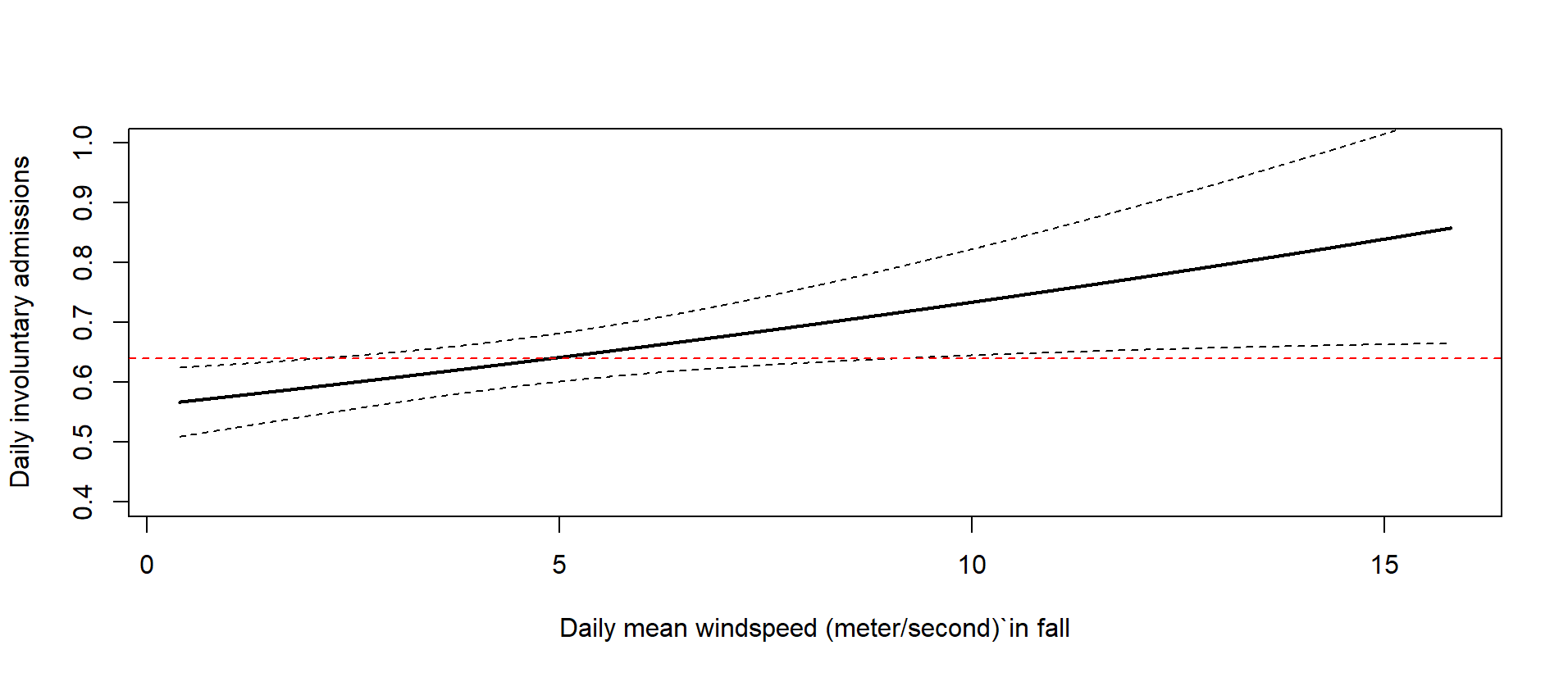  **B** |

The y-axes represent the expected daily numbers of involuntary admissions according to the Generalized Additive Models (GAMs) we used, averaged per psychiatric institution, and the x-axes the values of each significantly associated meteorological variable. The dotted black line represents the 95% confidence interval of the association and the dotted red line the mean daily involuntary admissions numbers. (A) all data analyses, (B) fall data

Supplementary Figure 8 – Association plots of lagged GAM analyses between mean ambient temperature and involuntary admissions

| 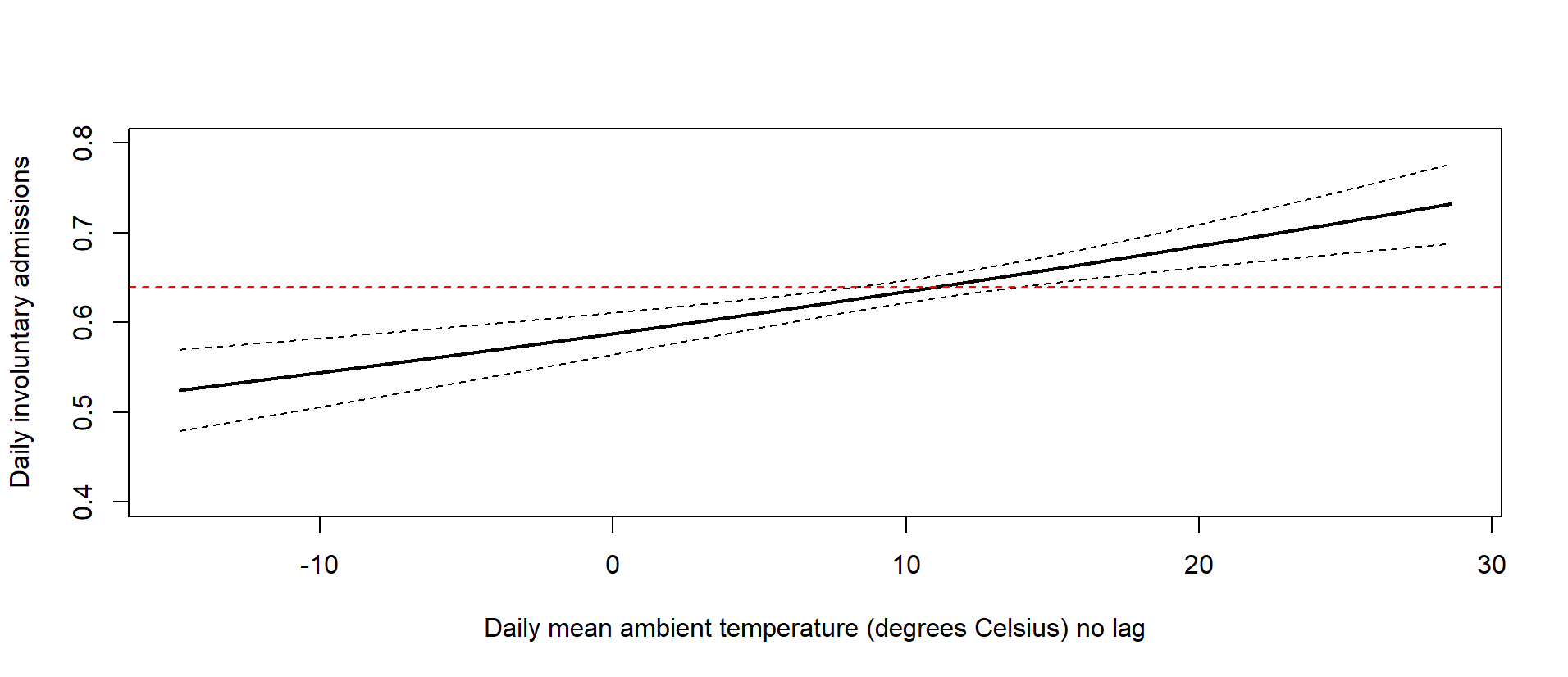 | 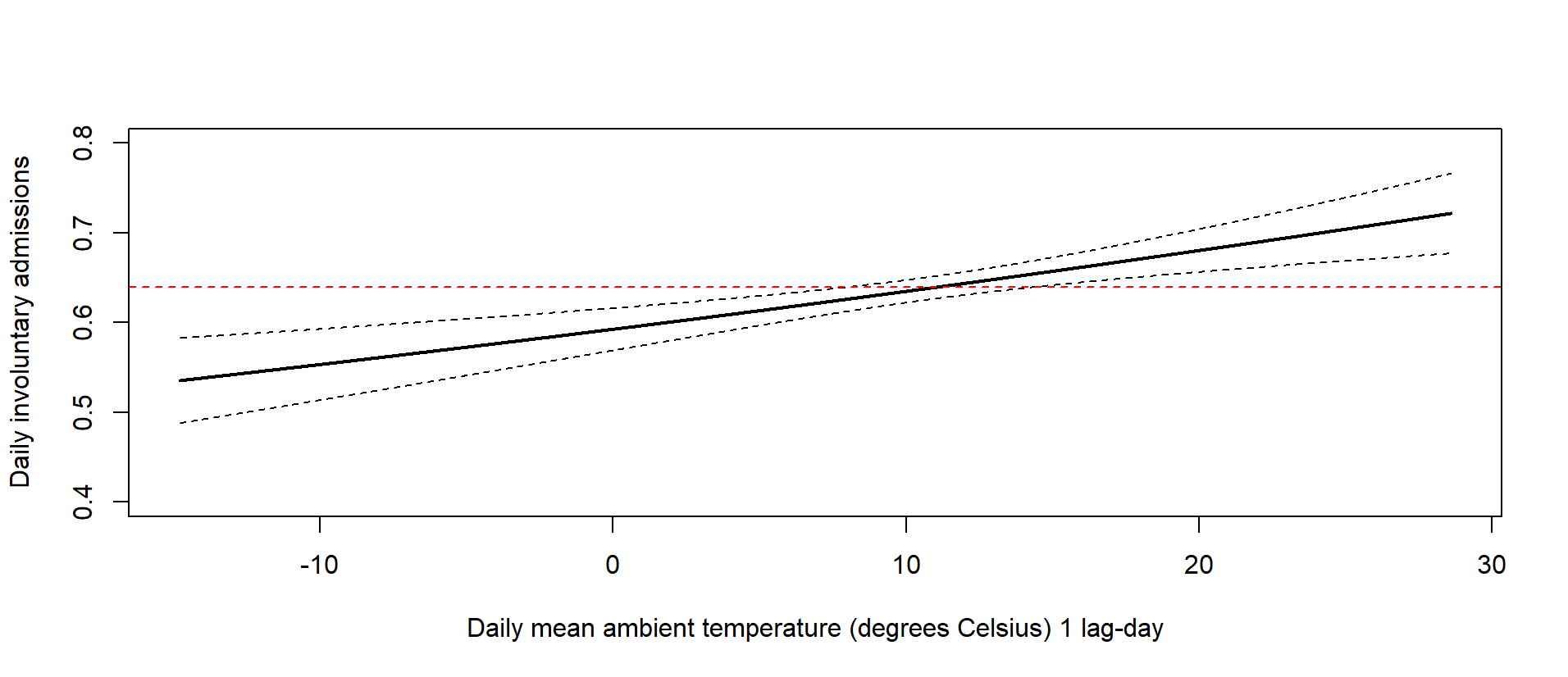  **A**  **B** |
| --- | --- |
| 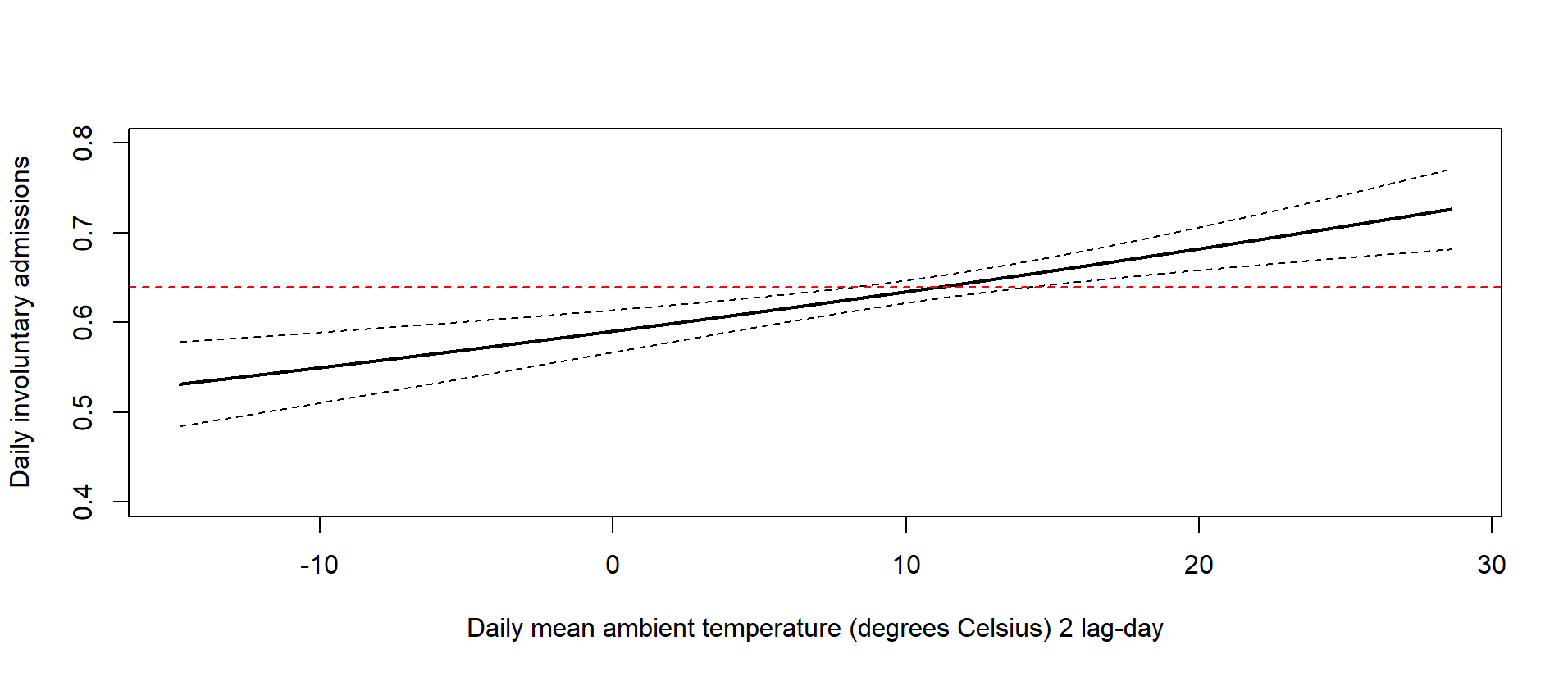  **C** | 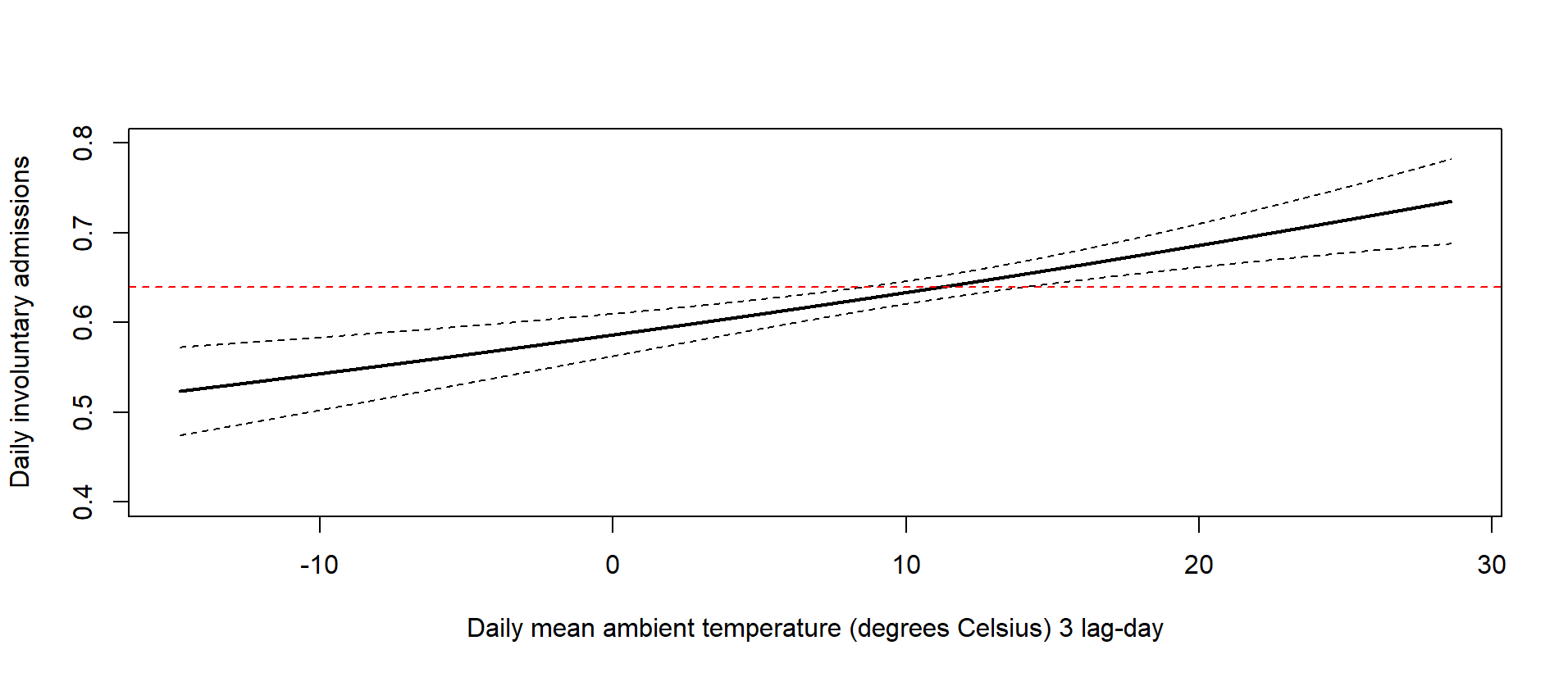  **D** |
| 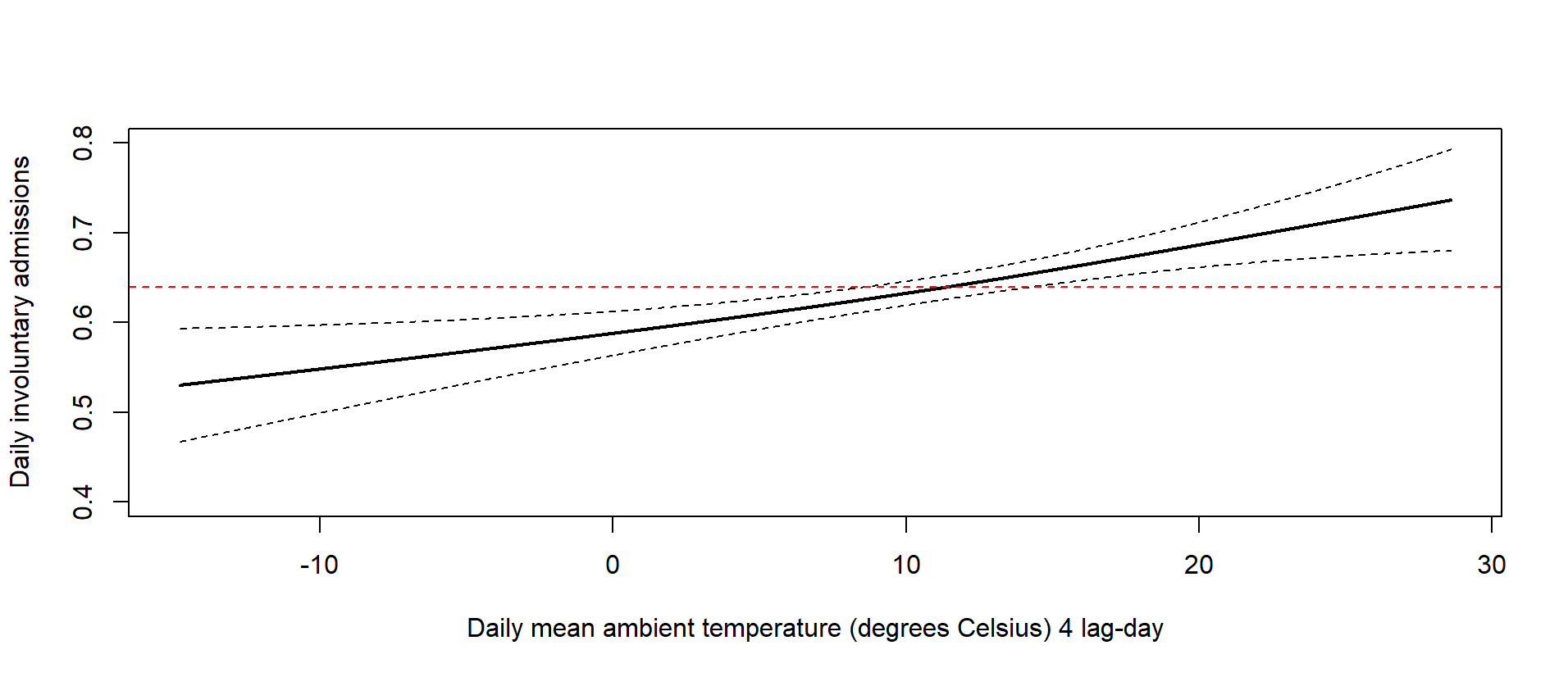  **E** | 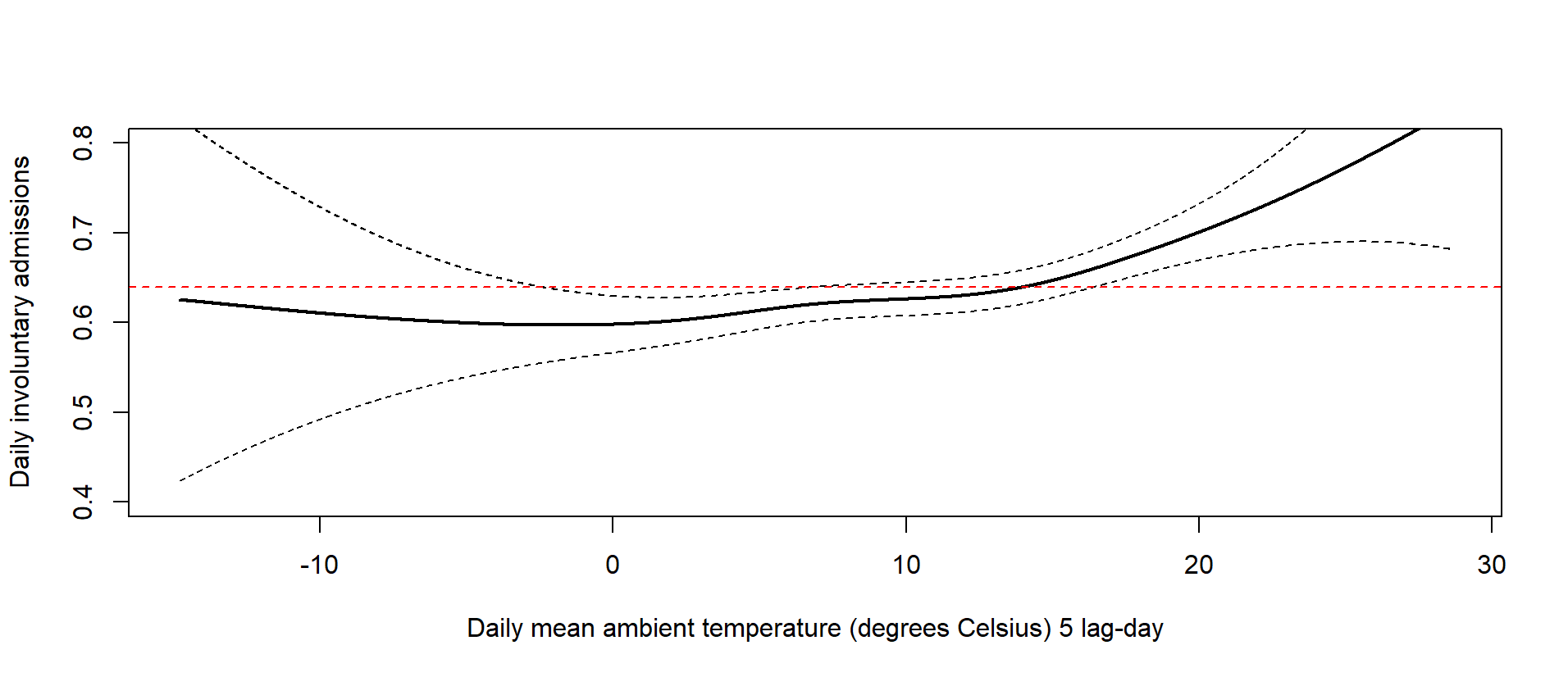  **F** |
| 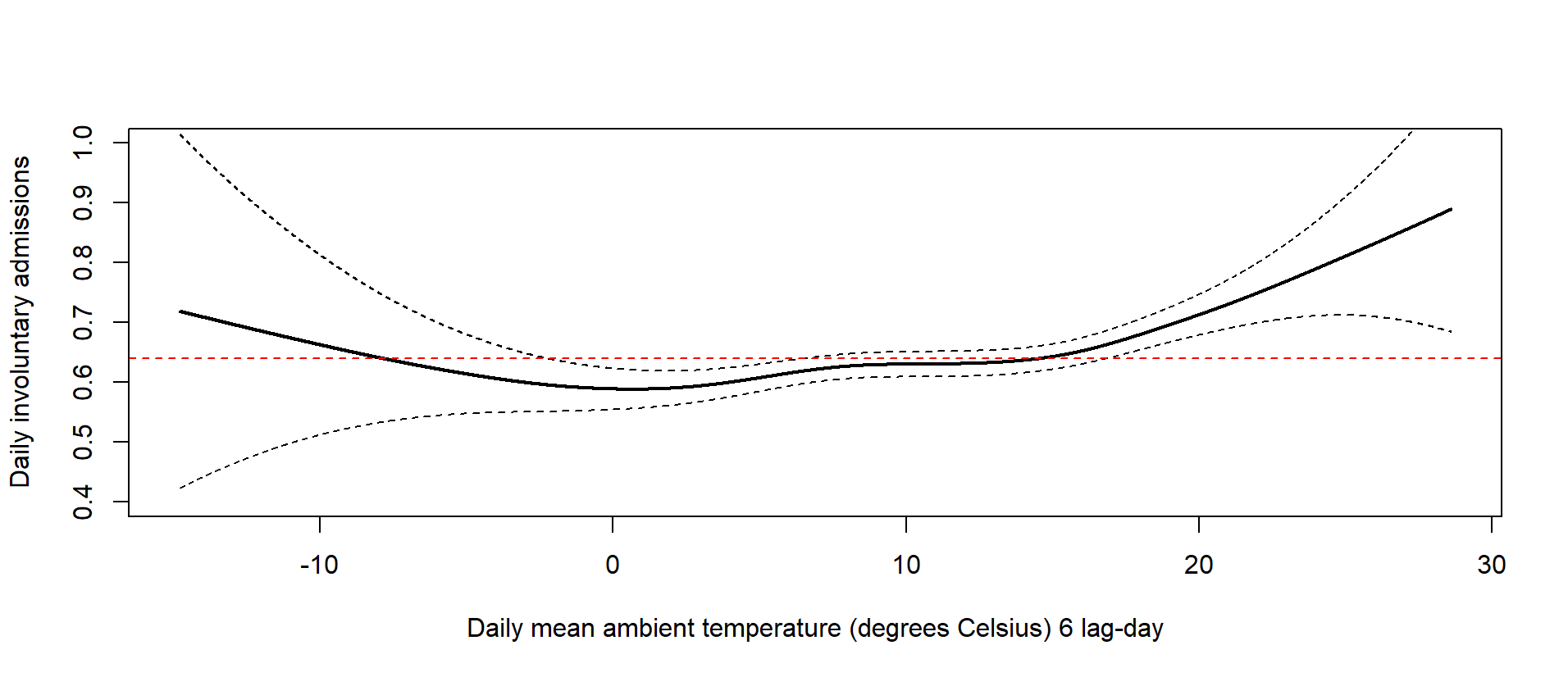  **G** | 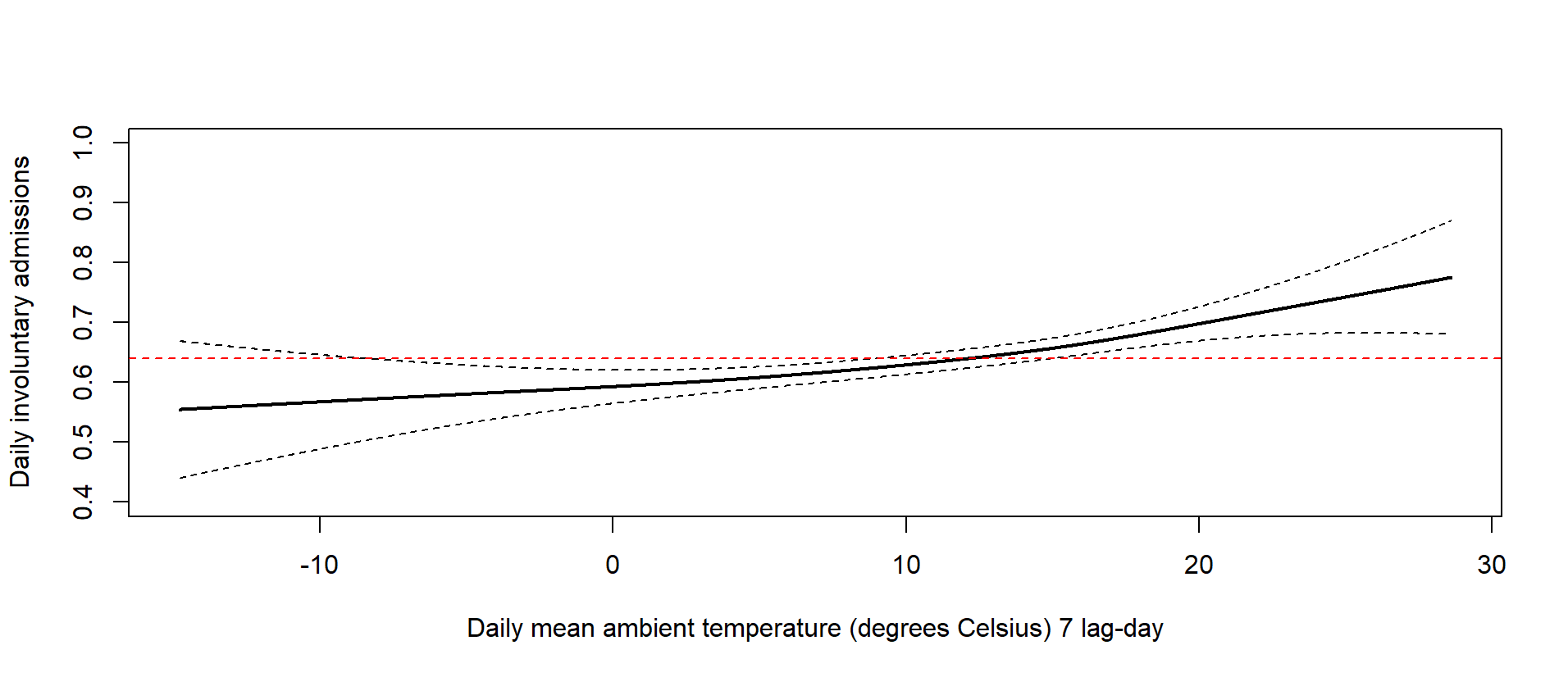  **H** |

Association plots of univariable GAM analyses for 1-7 days lagged mean ambient temperature variables. (A) no lag, (B) 1 day lag, (C) 2 days lag, (D) 3 days lag, (E) 4 days lag, (F) 5 days lag, (G) 6 days lag, (H) 7 days lag. The y-axes represent the expected daily numbers of involuntary admissions according to the Generalized Additive Models (GAMs) we used, averaged per psychiatric institution, and the x-axes the values of each significantly associated meteorological variable. The dotted black line represents the 95% confidence interval of the association, the dotted red line the mean daily involuntary admissions numbers

Supplementary Figure 9 – Lambda-estimation plot for temperature-binned involuntary admission case

| 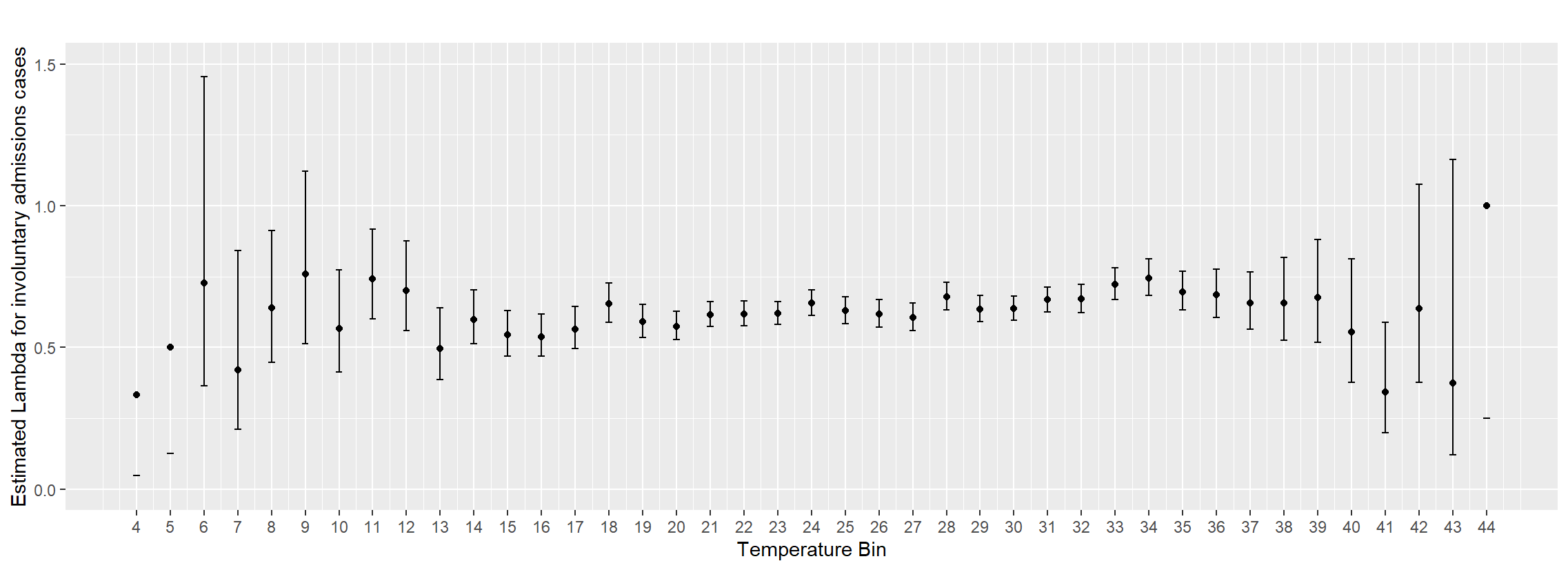 |
| --- |
| \| **Bin** \| **Minimum value (degrees Celsius)** \| **Maximum value (degrees Celsius)** \| \| --- \| --- \| --- \| \| 1 \| -14,8 \| -13,8 \| \| 2 \| -13,6 \| -13,2 \| \| 3 \| -12,7 \| -12,1 \| \| 4 \| -11,5 \| -10,8 \| \| 5 \| -10,7 \| -9,8 \| \| 6 \| -9,6 \| -8,8 \| \| 7 \| -8,7 \| -8,0 \| \| 8 \| -7,8 \| -6,9 \| \| 9 \| -6,8 \| -5,9 \| \| 10 \| -5,8 \| -4,9 \| \| 11 \| -4,8 \| -3,9 \| \| 12 \| -3,8 \| -2,9 \| \| 13 \| -2,8 \| -1,9 \| \| 14 \| -1,8 \| -0,9 \| \| 15 \| -0,8 \| 0,1 \| \| 16 \| 0,2 \| 1,1 \| \| 17 \| 1,2 \| 2,1 \| \| 18 \| 2,2 \| 3,1 \| \| 19 \| 3,2 \| 4,1 \| \| 20 \| 4,2 \| 5,1 \| \| 21 \| 5,2 \| 6,1 \| \| 22 \| 6,2 \| 7,1 \| \| 23 \| 7,2 \| 8,2 \| \| 24 \| 8,3 \| 9,2 \| \| 25 \| 9,3 \| 10,2 \| \| 26 \| 10,3 \| 11,2 \| \| 27 \| 11,3 \| 12,2 \| \| 28 \| 12,3 \| 13,2 \| \| 29 \| 13,3 \| 14,2 \| \| 30 \| 14,3 \| 15,2 \| \| 31 \| 15,3 \| 16,2 \| \| 32 \| 16,3 \| 17,2 \| \| 33 \| 17,3 \| 18,2 \| \| 34 \| 18,3 \| 19,2 \| \| 35 \| 19,3 \| 20,2 \| \| 36 \| 20,3 \| 21,2 \| \| 37 \| 21,3 \| 22,2 \| \| 38 \| 22,3 \| 23,2 \| \| 39 \| 23,3 \| 24,2 \| \| 40 \| 24,3 \| 25,2 \| \| 41 \| 25,3 \| 26,2 \| \| 42 \| 26,3 \| 27,1 \| \| 43 \| 27,3 \| 27,7 \| \| 44 \| 28,5 \| 28,6 \| |

(A) Graph with the estimated lambda (mean) values with a 95% confidence interval for temperature-binned involuntary admission cases, estimated with a Generalized Linear Model assuming Poisson distribution. Temperature bins with a mean involuntary admission value of ‘0’ were excluded for the estimation model to function. (B) shows the minimum and maximum values in the 1 degrees Celsius wide bins for temperature

Bibliography

Brooke Anderson, G., Bell, M. L., & Peng, R. D. (2013). Methods to calculate the heat index as an exposure metric in environmental health research. In *Environmental Health Perspectives* (Vol. 121, Issue 10). https://doi.org/10.1289/ehp.1206273

Geert Groen. (2009). *Windchill equivalent temperature (WCET) : climatology and scenarios for Schiphol Airport*.

Koninklijk Nederlands Meteorologisch Instituut. (2000). *Handbook for the Meteorological Observation*.
